# A clinical protocol for the detection of comorbidities associated with monogenic causes of male infertility

**DOI:** 10.1093/humrep/deag038

**Published:** 2026-03-21

**Authors:** G W van der Heijden, D Westra, M C S van Olden, W Hobo, M S Oud, R M Smits, Ö Baysal, H Bogers, K D’Hauwers, M J E Kempers, J F M Jacobs, J A Veltman, M F Stokman, L Ramos

**Affiliations:** Department of Obstetrics and Gynecology, Radboud University Medical Center, Nijmegen, The Netherlands; Department of Human Genetics, Radboud University Medical Center, Nijmegen, The Netherlands; Department of Ophthalmology, Radboud University Medical Center, Nijmegen, The Netherlands; Laboratory Medical Immunology, Department of Laboratory Medicine, Radboud University Medical Center, Nijmegen, The Netherlands; Laboratory of Hematology, Department of Laboratory Medicine, Radboud University Medical Center, Nijmegen, The Netherlands; Department of Human Genetics, Radboud University Medical Center, Nijmegen, The Netherlands; Department of Obstetrics and Gynecology, Radboud University Medical Center, Nijmegen, The Netherlands; Department of Human Genetics, Radboud University Medical Center, Nijmegen, The Netherlands; Department of Obstetrics and Gynecology, Radboud University Medical Center, Nijmegen, The Netherlands; Department of Urology, Radboud University Medical Center, Nijmegen, The Netherlands; Department of Human Genetics, Radboud University Medical Center, Nijmegen, The Netherlands; Laboratory Medical Immunology, Department of Laboratory Medicine, Radboud University Medical Center, Nijmegen, The Netherlands; Institute of Genetics & Cancer, College of Medicine and Veterinary Medicine, University of Edinburgh, Edinburgh, UK; Department of Human Genetics, Radboud University Medical Center, Nijmegen, The Netherlands; Department of Obstetrics and Gynecology, Radboud University Medical Center, Nijmegen, The Netherlands

**Keywords:** male infertility, infertility, comorbidity, MEI1, DNAH17, Human Protein Atlas, deep phenotyping, monogenic, genetics

## Abstract

**STUDY QUESTION:**

How can comorbidities associated with monogenic forms of male infertility systematically be identified?

**SUMMARY ANSWER:**

Via a framework that consists of seven sequential steps, gene-specific phenotyping protocols can be generated for all monogenic causes of male infertility.

**WHAT IS KNOWN ALREADY:**

Infertility negatively impacts men’s health. When loss of a single gene is causal for infertility (i.e. monogenic), the associated comorbidities are largely unknown.

**STUDY DESIGN, SIZE, DURATION:**

A framework was developed that allows the generation of gene-specific phenotyping protocols. The framework was applied to generate such protocols for two men, each with a different monogenic cause for their infertility.

**PARTICIPANTS/MATERIALS, SETTING, METHODS:**

A multidisciplinary medical team formulated seven sequential steps to develop gene-specific phenotyping protocols. Gene-specificity was obtained by using a gene’s expression pattern in the human body to identify tissues/cell types that show high levels of expression, as well as a literature search on the gene of interest, for potential morbidities. With these insights, tailored questionaries and tests are designed. We applied this framework to generate gene-specific protocols for two men in whom infertility was caused by the respective disruption of the genes MEI1 and DNAH17. These genes respectively show very high levels of expression in various types of immune cells and retinal cells. With gene-specific phenotyping protocols, we assessed the functionality of these cells in these men.

**MAIN RESULTS AND THE ROLE OF CHANCE:**

Our framework facilitated the generation of two gene-specific phenotyping protocols that were used to systematically identify potential comorbidities associated with two forms of monogenic male infertility. Analyses of the gene expression in the human body identified immune cells (MEI1) and retinal cells and oligodendrocytes (DNAH17) as somatic cell types with high expression. Gene-specific phenotyping protocols contained targeted questions as well as clinical tests for these tissues/cell types. The questionnaires indicated no increased susceptibility to infections, allergies nor autoimmune disease (MEI1) or visual problems (DNAH17). The clinical tests comprised extensive immune profiling for the MEI1-participant and functional evaluation and imaging of the retinal cells of the DNAH17-participant. None of the test results indicated clinically relevant alterations at present. To identify true comorbidities, or lack thereof, more men with the same monogenic cause should be phenotyped.

**LARGE SCALE DATA:**

Not applicable.

**LIMITATIONS, REASONS FOR CAUTION:**

The Human Protein Atlas database was used to assess the expression pattern of the causal gene. This database only contains expression data in adult tissues. Potential comorbidities due to a developmental function of a gene can therefore be missed. In addition, comorbidities might develop later in life and might not be present during the phenotyping.

**WIDER IMPLICATIONS OF THE FINDINGS:**

Knowledge on the presence or absence of infertility-associated comorbidities allows clinicians to counsel patients on possible additional health risks for themselves and potential future offspring. As a consequence, medical care for infertile people extends beyond reproductive needs to general health.

**STUDY FUNDING/COMPETING INTEREST(S):**

J.A.V. was funded by an Investigator Award in Science from the Wellcome Trust (209451) and by The Netherlands Organization for Scientific Research (918-15-667). The authors declare no conflicts of interest.

**TRIAL REGISTRATION NUMBER:**

N/A.

## Introduction

In humans, reproductive fitness appears to be an indicator of general health (Punjani and Lamb, 2020; Burke *et al.*, 2022; Zhao *et al.*, 2024). For males, a direct correlation between semen quality and life expectancy has been reported (Eisenberg *et al.*, 2014; Priskorn *et al.*, 2025). Epidemiological studies that assessed general health issues in heterogeneous cohorts of ‘infertile men’ also indicated such a link: male infertility was found to be associated with a higher incidence of premature mortality and increased morbidity (Glazer *et al.*, 2017, 2019; Del Giudice *et al.*, 2020, 2021; Hansen *et al.*, 2023). The precise disease mechanisms at work are still largely unclear though main actors are likely altered hormone levels, mental distress, and genetics. Elucidating the complex interplay between these factors has been named a top priority for future infertility research (Duffy *et al.*, 2021).

Interestingly, the correlation between reproductive fitness and general health seems to be dose-dependent: the less spermatozoa men produce the higher the chances of health issues (Priskorn *et al.*, 2025). This is possibly linked to the observation that the most severe form of male infertility, namely spermatogenic failure, in which the production of spermatozoa is halted or severely reduced, frequently has a genetic cause (Krausz and Riera-Escamilla, 2018). Since an infertility-causing genetic defect is often present in all cells of the body, the potential for other health issues (comorbidities) to develop exists. For example, in the case of Klinefelter syndrome, in which an additional X-chromosome causes infertility, the presence of two X-chromosomes (next to a Y chromosome) in somatic cells causes several comorbidities due to increased gene dosage and changes in the activity of protein interactomes (Belling *et al.*, 2017). Additionally, multiple genes essential for fertility are involved in processes like DNA repair. Loss of their functionality in germ cells causes infertility, while simultaneously it predisposes to various types of cancer (Punjani and Lamb, 2020; Wyrwoll and Steingröver, 2024). Interestingly, the load of cancer-linked variants is increased in infertile men (Valkna *et al.*, 2025).

The relationship between male reproductive fitness and general health could thus, in part, be explained by genetics. This notion raises the importance of understanding the genetics of male infertility, since these genetic factors might be responsible for more than fertility impingement alone (Veltman and Tüttelmann, 2024). In the last years, our understanding of the genetics of male infertility has grown rapidly (Oud *et al.*, 2019, 2022; Nagirnaja *et al.*, 2022; Lillepea *et al.*, 2024; Riera-Escamilla and Nagirnaja, 2025). Overall, the genetic causes are numerous, ranging from chromosomal scale (Klinefelter, XYY, and translocations) or deletions (Y-chromosome) that affect numerous genes, to loss of function of a single gene (Krausz and Riera-Escamilla, 2018; Tüttelmann *et al.*, 2018; Houston *et al.*, 2021). With many genes that still need to be identified as essential for fertility, this latter category makes up the dark matter of the genetic causes of male infertility. Currently, the number of genes that have a gene–disease association strong enough to be included in a diagnostic gene-panel for male infertility is around 130 (Oud *et al.*, 2025), a number that is rapidly increasing. A conservative estimation of the total number of genes that, when mutated, cause azoospermia is ∼600 (Nagirnaja *et al.*, 2022). A number that is even higher when other infertility-causing sperm abnormalities are considered (Wyrwoll *et al.*, 2024).

Since fertility genes, defined by their necessity for fertility, are frequently also expressed outside the gonadal tissues, their loss, or diminished gene function can also affect other tissues. This in turn could cause comorbidities. It is expected that infertile men who share a monogenic cause will have a variable degree of overlapping comorbidities (Stessman *et al.*, 2014).

The identification of comorbidities through a general assessment of a patient’s health is common practice in the field of clinical genetics and is also known as phenotyping. Essential for comorbidity identification is the use of so-called ‘deep phenotyping’ (Robinson, 2012). This is defined as the systematic, precise, and comprehensive analysis of phenotypic abnormalities in which the individual components of the phenotype are observed and described for the scientific examination of human disease. The combination of genetic data and deep phenotyping has led to a diagnostic approach known as ‘reversed phenotyping’ or the ‘genotype-first approach’ (Stessman *et al.*, 2014). The principle of reversed phenotyping is that patients are screened for specific associated phenotypes based on the outcome of genetic testing (Stessman *et al.*, 2014). Several studies have used this approach to characterize novel syndromes and understand the comorbidities accompanying the ‘main’ disease (Akawi *et al.*, 2015; Vissers and Veltman, 2015), for example the frequent occurrence of obesity in patients with intellectual disability based on a mutation in the *PHIP* gene (de Goede *et al.*, 2016; Jansen *et al.*, 2018).

To identify potential comorbidities in monogenic severe male infertility, reversed phenotyping can offer a starting point. However, since the biological functions and expression patterns of fertility genes are widely variable, so can be the associated phenotype. Loss or diminished function of a fertility gene will potentially cause a gene-specific set of health issues. This warrants a deep phenotyping approach for each individual fertility gene.

For deep phenotyping, a standardized questionnaire can be used that gathers information on the subjects’ medical, social, childhood, and familial histories as well as a physical examination. Here, we describe how gene-specific phenotyping protocols can be developed and applied. We propose that the gene expression pattern as well as notions from scientific literature on the gene of interest can be used to formulate additional questions and medical tests. A multidisciplinary medical team (MMT) can weigh the burden and benefit of these, and questions and tests can be incorporated into the general protocol to generate a gene-specific deep phenotyping protocol. We applied this approach to two men with monogenic infertility caused by mutations in *MEI1* and *DNAH17*, both of which are also highly expressed in specific somatic cell types.

## Materials and methods

### Ethical approval

The subjects in this study visited the Department of Obstetrics and Gynecology of the Radboud University Medical Center during 2023–2024. The study protocol was approved by the Ethics Committee Nijmegen: NL50495.091.14 version 6.0. For both patients, written informed consent from the patient was obtained prior to enrollment in the study.

### Role and composition of MMT

**Figure 1.**
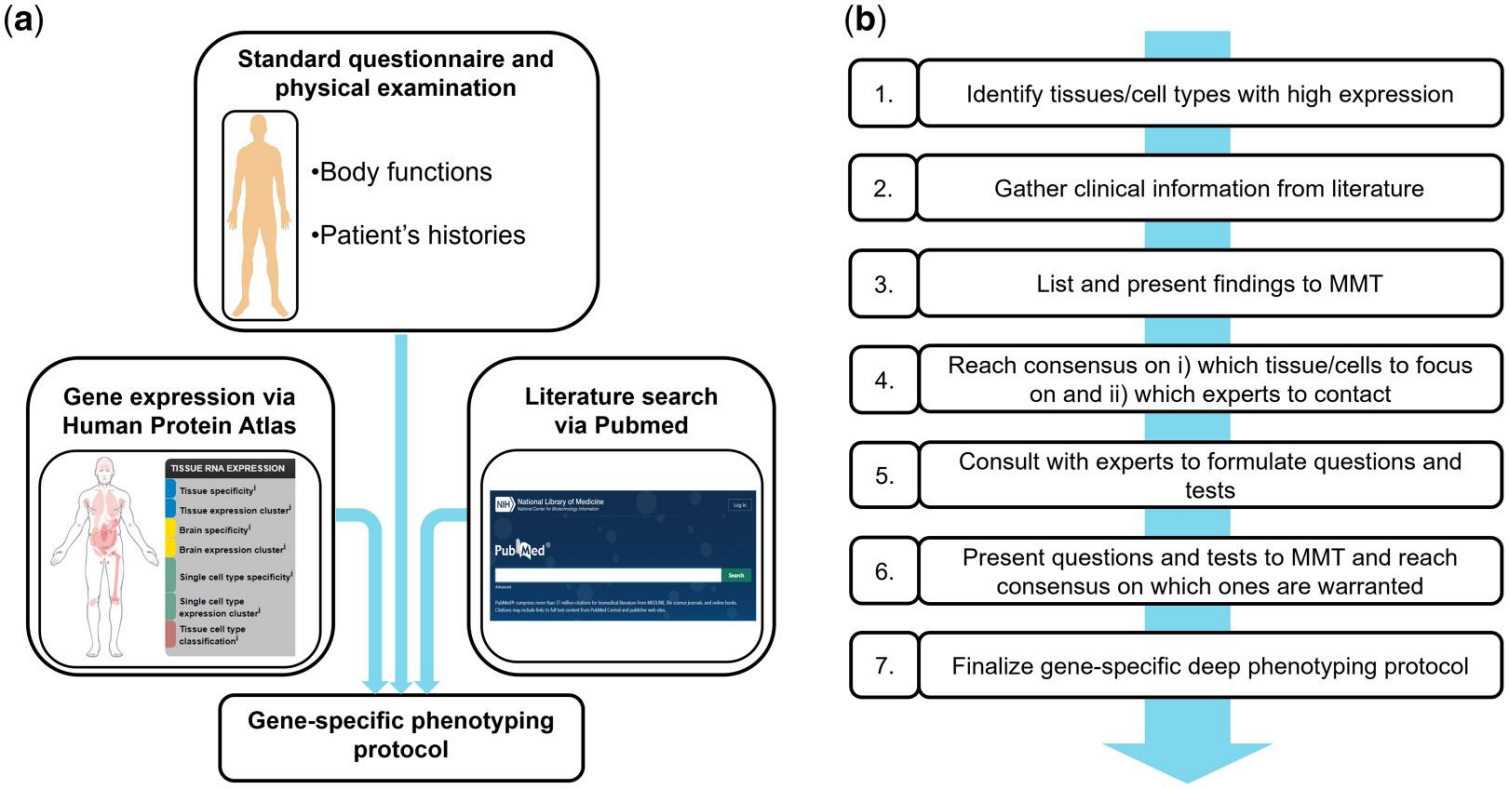
**Schematic overview of the data sources of a gene-specific phenotyping protocol and the seven steps for its development.** (**a**) To produce a gene-specific phenotyping protocol, a general phenotyping protocol is supplemented with targeted questions based on the gene expression pattern in the human body and clinical observations derived from a literature search. For the former, the Human Protein Atlas can be used, for the latter Pubmed. (**b**) The development of a gene-specific phenotyping protocol in seven steps. MMT, multidisciplinary medical team.

**Figure 2.**
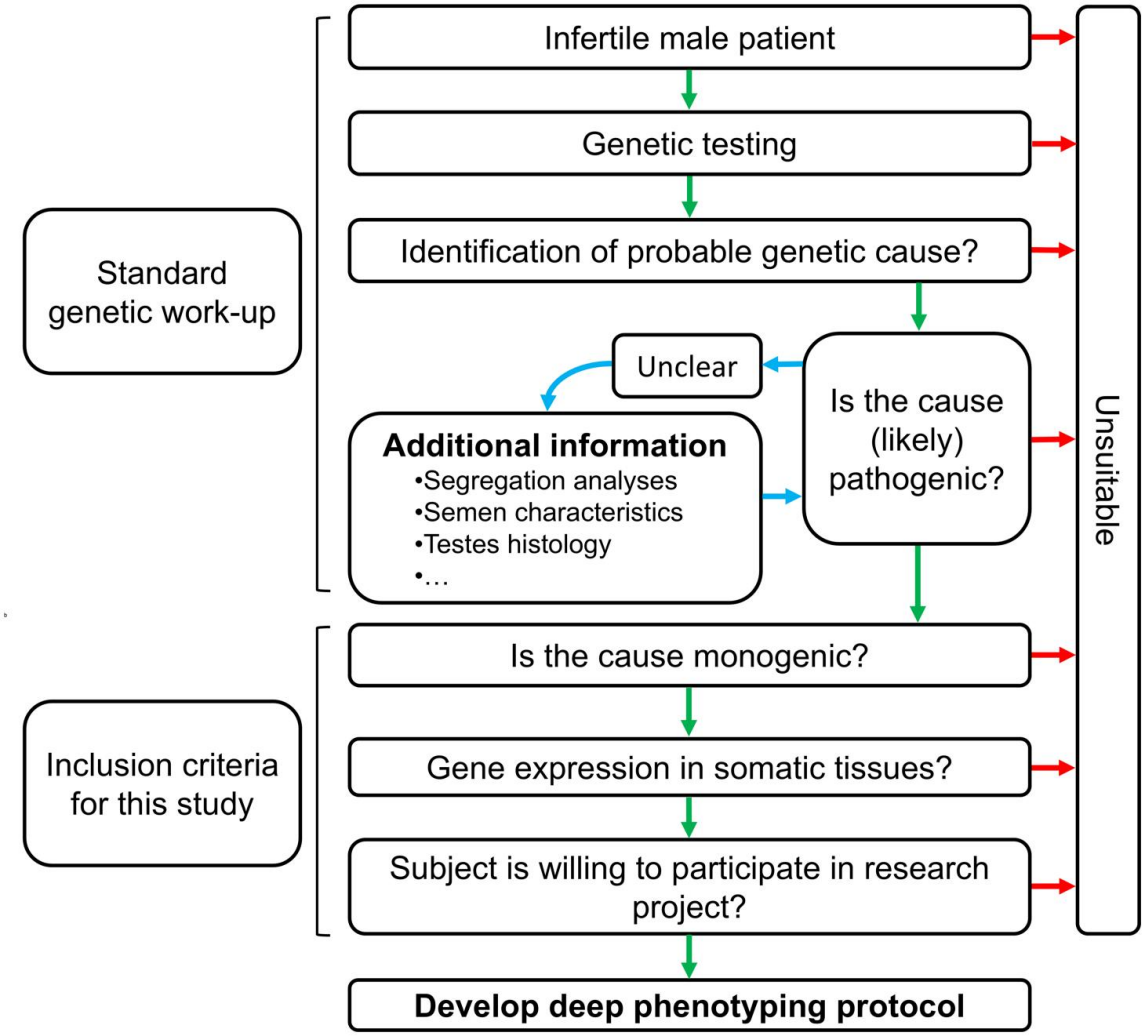
**Flow diagram with requirements for participation in this study.** (**a**) Description of all steps as part of the genetic diagnostic work up. (**b**) major inclusion requirements for this study. Green arrows present ‘yes’, red ones ‘no’, blue ones correspond to intermediate steps.

To guide the process of medical research protocol development, we decided to assemble an MMT. The MMT consisted of two clinical geneticists, who both regularly assess patients’ phenotypes as part of their clinical practice, a urologist with a certification for clinical andrology provided by the European Association of Andrology, a gynecologist specialized in reproductive medicine, a clinical laboratory geneticist, certified by the European Board of Medical Genetics, and two senior clinical embryologists, both certified by the European Society of Human Reproduction and Embryology. At first, the MMT focused on the preliminary work of (i) developing a general strategy to generate gene-specific phenotyping protocols (Fig. 1) and (ii) the requirements for inclusion in this study (Fig. 2). Next, the MMT discussed draft versions of gene-specific phenotyping protocols. Specific questions and medical tests that were proposed to be added to the general phenotyping protocol were evaluated and discussed. Decisions were made unanimously.

### Inclusion criteria

The standard genetic diagnostic work-up of our male patients with severe infertility includes an exome sequencing-based gene panel for the detection of monogenic causes and copy number variants, as previously described (Oud *et al.*, 2025). Only genes with an established gene–disease relationship are included in the gene panel. Variants are interpreted via strict adherence to the American College of Medical Genetics and Genomics/Association for Molecular Pathology (ACMG/AMP) guidelines (Richards *et al.*, 2015). Only likely pathogenic and pathogenic variants are considered as (likely) causal. In case of recessive disease, segregation analysis is performed to confirm compound heterozygosity. When Variants of Unknown Significance (VUS) are found, additional tests including semen characteristics or testis histology can be used to allow a better interpretation of these variants and potentially a reclassification to (likely) pathogenic (Fig. 2). For this study only patients with a (likely) pathogenic variant(s) in a gene that shows expression outside the testes and/or has been associated with other phenotypes in addition to infertility were included. In case (likely) pathogenic variants are found in more than one of the assessed genes, the patient is excluded from this study.

### Andrological evaluation of participants

Both men were referred to the Department of Obstetrics and Gynecology of the Radboud University Medical Center with their partner with a wish to conceive. Prior knowledge of male cause for infertility was present based on a semen analysis elsewhere. Briefly, the andrological work-up is performed by a trained andrology specialist and consists of a semen analyses, an endocrine evaluation, assessment of the urological history, assessment of the urogenital tract (scrotum’s form, presence and consistency of the testes, epididymis and vas deferens, form and length of the penis, and position of the meatus). When indicated (anomaly of vas deferens) a sonogram of the kidneys is performed. Furthermore, potential problems with micturition, sexological issues, and pain in the genital region are discussed.

### Assessment of association between gene expression and comorbidities

The expression pattern for genes identified as causal for syndromic male infertility (n = 17) (Houston *et al.*, 2021) was determined via the Human Protein Atlas (HPA). For each of the 17 genes, the organs/cell types with the highest levels of expression were taken (Supplementary Data Files S1 and S2). Separately, for each gene, the reported comorbidities as reported in OMIM were listed. Comorbidities with a shared underlying cause were grouped as one. For example: in most primary ciliary dyskinesia syndromes infections in the nose, bronchus, lung, and ear stem from impaired ciliary clearance were grouped. Comorbidities in organs for which the HPA does not have expression data (e.g. the ear) were disregarded. Since the HPA expression data are generated from tissues derived from adults, congenital comorbidities were also disregarded.

### Selection of tissues/cell types for comorbidity screening

To determine gene expression patterns and select the tissues/cell types for morbidity screening, the gene of interest was queried via the HPA portal (Karlsson *et al.*, 2021). In the ‘Summary’ tab, the sub-tabs ‘tissue RNA expression’ and ‘cell type RNA expression’ are located (Supplementary Data File S1). The former presents integrated datasets of bulk RNA sequencing expression data while the latter presents data derived from single-cell RNA sequencing. From the ‘tissue RNA expression’ tab, all tissues listed under ‘Tissue specificity’ were taken and from the ‘cell type RNA expression’ tab all cell types listed under ‘Single cell type specificity’ were taken. These tissues and cell types all show a relative expression level at least 4-fold higher than the mean expression of all tissues/cell types for the queried gene. This cut-off is set by the HPA.

### Immune profiling for *MEI1* mutation

Immunological tests were performed in routine and specialized diagnostics laboratory of the Radboudumc (ISO15189 accredited). Total IgG, IgA, IgM, and IgD were measured using turbidimetry on a Cobas 6000 (Roche, Basel, Switzerland). Measurements were performed according to manufacturer guidelines. Total IgE was measured on an Immunocap 250 (Thermo Fisher Scientific, Uppsala, Sweden). IgG subclasses were measured using nephelometry on an Atellica (Siemens Healthineers, Erlangen, Germany). Antinuclear antibody profiling was performed with indirect immunofluorescence on Hep-2 cells (Inova Diagnostics, San Diego, USA). M-protein screening was performed with serum protein electrophoresis and immunofixation electrophoresis on a Hydrasys2 (Sebia, Lisses, France). Immune-phenotyping analysis was performed on fresh EDTA blood by using flow cytometry as previously described (Huys *et al.*, 2022). Briefly, the frequencies and absolute counts of the main lymphocyte subsets (CD3+ total T cells, CD3+CD4+ T-helper cells, CD3+CD8+ cytotoxic T cells, CD19+ B cells, and CD3-CD56+ NK cells) were determined using an automated Aquios Tetra assay (Beckman Coulter, Woerden, The Netherlands). In addition, the activation, differentiation, and subset composition of T cells, B cells, and NK cells were evaluated using validated laboratory-developed tests on cells subjected to bulk lysis followed by specific stainings and measurement on a Navios flow cytometer (Beckman Coulter). Data were analyzed with Kaluza software (Beckman Coulter).

### Profiling of the retina for *DNAH17* mutation

All assessments were conducted during a single-day visit. All tests and imaging procedures were performed in both eyes. Color vision was evaluated using the Hardy–Rand–Rittler test. Refraction was measured, followed by best-corrected visual acuity using a 4-m ETDRS chart. Axial length was measured with the IOLMaster 700 (Carl Zeiss Meditec AG, Jena, Germany). Fundoscopy was performed through slit-lamp examination. Fundus photography was conducted using the Topcon DRI OCT Triton. Fundus autofluorescence (FAF) and spectral-domain optical coherence tomography (SD-OCT, dense volume scan: 30° × 25°, 241 sections, ART = 9, high resolution) were acquired with the Heidelberg Spectralis (Heidelberg Engineering, Germany). Static perimetry was conducted using the Octopus 900 (Haag-Streit, Switzerland) with EyeSuite software (v i8.2.2.0/perimetry v3.6.1), employing the OP3/32 Dynamic White-White protocol. Electroretinography (ERG) was performed using the Espion e3 system (Diagnosys LLC, UK) with Dawson, Trick, and Litzkow (DTL) electrodes, including scotopic (V5), photopic (V6.1), and multifocal ERG (61 hexagons), in accordance with the standards of the International Society for Clinical Electrophysiology of Vision (ISCEV). A dark adaptation test was also performed using the same system. Finally, adaptive optics imaging (AO-FIO, ImagineEyes™, Orsay, France) was used to evaluate photoreceptor structure and cone density.

### Electron microscopy

Washed spermatozoa were fixed overnight in 2% Glutaaraldehyde in 0.1M Na-cacodylatebuffer. After washing in cacodylaatbuffer cells were fixed for 1 h in 2% OsO_4_. Pelleted cells were subsequently prepared in agar, dehydrated, and placed in epon. Sections were cut at a thickness of 60 nm and collected on a 200-mesh copper grid.

## Results

### Design of a framework for gene-specific phenotyping protocol development

Our goal was to design a framework that facilitates the development of gene-specific phenotyping protocols. Ease-of-use for any clinical geneticist interested in a comorbidity assessment was a key requirement. As a departure point, we leveraged the fact that deep phenotyping of patients, aimed to identify comorbidities, is a routine part of the clinical genetic practice. The core structure of our proposed phenotyping protocol is therefore based on the standard phenotyping workflow of our in-house clinical geneticists. It consists of the following topics: a thorough review of current health problems, medical history, perinatal and childhood development, family history including a pedigree of the family, assessment of comorbid medical conditions with focus on chronic diseases such as cardiovascular disease or diabetes, and a full physical examination including the examination of dysmorphic features (Supplementary Data File S3).

Next, we adapted this general core protocol into a gene-specific one. It is a well-accepted notion that the expression levels of a gene in a tissue or cell type, often reflects the gene’s functional importance in those contexts. For example: the increased expression levels of the Rhodopsin gene (*RHO*) in rod photoreceptor cells reflects its essential role in vision. We explored if gene expression levels could also be used as a proxy for gene relevance in the context of male infertility. To this end, we analyzed 17 genes linked to a monogenic syndromic form of male infertility, in which infertility occurs alongside other comorbidities (see Materials and Methods and Supplementary Data File S2). For each syndrome, we determined whether organ/cell types with high gene expression overlapped with the anatomical sites of the other comorbidities. For 12 of the 17 genes, the expression data identified an organ/cell type that corresponded to the site of morbidity. When considering all comorbidities across the 17 syndromes (n = 29, Materials and Methods), ∼40% overlapped with organs/cell types identified as high expression (n = 12). Based on these findings, we conclude that expression data can be a valuable source of information to identify potential sites of morbidities in the human body. For the identification of the tissues/cell types with the highest levels of expression of a fertility gene of interest, we applied the same strategy as described above (see Materials and Methods). To limit the number of organs/cell types for morbidity follow-up, we choose to only select the ones listed in the HPA as the tissues/cell types with at least a 4-fold expression of the mean (Supplementary Data Files S1 and S2).

We also considered literature published on the gene of interest as an additional data source for specifying phenotyping protocols. This body of literature should be assessed for clinical observations that could hint at morbidities. When the number of articles is too vast to digest, additional terms like ‘human’ or ‘clinical’ can be used to narrow down the search.

### Generation of a gene-specific phenotyping protocol in seven steps

After defining the components of a gene-specific phenotyping protocol, we worked out a standardized approach to produce such a protocol. This procedure consists of seven steps (Fig. 1b) and starts with data gathering. Gene expression data are obtained from the HPA (step 1), and clinically relevant observations are identified through a literature search (step 2). The data produced during these searches should be collated into an easily digestible format. Next (step 3) these findings should be presented to the MMT. Consensus should be reached on which tissues/cells and/or clinical observations deserve attention. Additionally, clinical experts can be identified to be contacted for their expertise (step 4). The contacting and involvement of the experts follows (step 5). With this input, targeted questions can be formulated that will help to determine if comorbidities are present. Standardized Human Phenotype Ontology (HPO) terms are used when possible. In addition, the expert can aid in suggesting medical tests that, too, would identify comorbidities. These questions and medical tests should then be presented and discussed within the MMT with the aim to identify the questions and tests that should be incorporated in order to create the gene-specific phenotyping protocol (step 6). Lastly (step 7), the gene-specific phenotyping protocol can be finalized and used for application.

### Generation of a *MEI1*-specific phenotyping protocol

**Figure 3.**
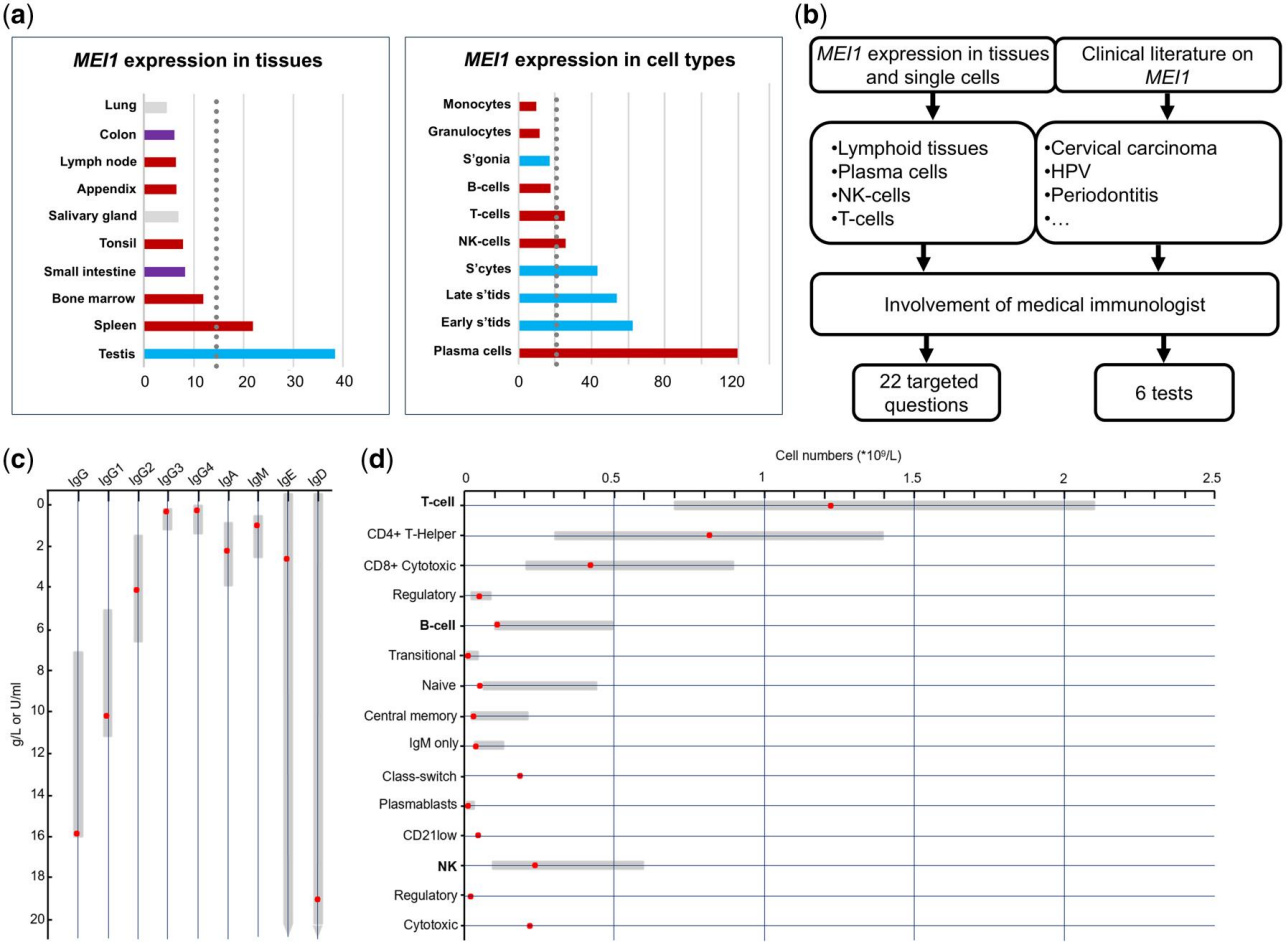
***MEI1* expression, protocol development and immune phenotyping.** (**a**) Top 10 tissues (left) and cell types (right) with highest relative expression of *MEI1*. Gray-dotted line corresponds with 4-fold mean. Bar coloring: blue for gonadal tissue or cells; red for immune-related tissues or cells, purple for tissues of the digestive tract, gray for other tissues. Labeling abbreviations: s’gonia: spermatogonia, s’cytes = spermatocytes, s’tids = spermatids. (**b**) Diagram summarizing the development of a *MEI1*-specific phenotyping protocol. (**c**) Concentration or activity of all immunoglobulin classes in the blood of subject 1. None of these have a concentration or activity outside the reference values. Red dot represents the measured values, gray bar the reference values. (**d**) Absolute number of immune cells in the blood of subject 1. None of the immune cell types evaluated were present in numbers that are clinically concerning. Red dot represents the measured values, gray bar the reference values.

Subject 1 was referred to our fertility clinic for an unfulfilled child wish. Routine diagnostic work-up indicated non-obstructive azoospermia. Genetic testing showed one pathogenic variant and a VUS in the *MEI1* gene (Supplementary Data File S4). Loss of *Mei1* in male mice results in a failure of the homologous chromosomes to properly synapse, causing a meiotic arrest (Libby *et al.*, 2002). The segregation analyses of the parents showed these variants to be bi-allelic. In addition, an analysis of the testis histology showed a meiotic arrest. This arrest type is typical for men with MEI1 loss-of-function (Ben Khelifa *et al.*, 2018). These additional data allowed the upgrading of the VUS to a likely pathogenic variant (Fig. 2). We therefore concluded that the identified *MEI1* variants are likely the cause of subject’s 1 infertility. Next, we assessed whether *MEI1* shows expression outside the gonadal tissues/cell types. This is indeed the case: the HPA reports abundant expression in immune-related tissues and cell types (Fig. 3a). We therefore developed an *MEI1*-specific phenotyping protocol according to the seven steps we described above (Supplementary Data Files S5 and S6). This protocol consists of 71 question/attentions points of which 21 are *MEI1*-prompted. In addition, it also contains six test panels aimed to phenotype the adaptive immune response (Supplementary Data File S7).

### Deep phenotyping results of *MEI1* subject

Overall, the subject’s answers indicated only minor medical issues as frequent ear infections during childhood (data not shown). Virtually all questions addressing the presence of symptoms that indicated an immunological disorder were answered negatively. This is in line with the laboratory results of the immune profiling. All the immunoglobulin levels were within or close to the reference values (Fig. 3c), suggesting normal functionality of the plasma cells. The screening for auto-antibodies was negative and no monoclonal proteins were detected. Total white blood cell count was 5.2 × 10^9^/L (reference range 3.7–10.7 × 10^9^/L). Analysis of the lymphocyte fractions showed no abnormalities including normal CD4+/CD8+ T-cell ratio, normal distribution of naïve and effector/memory T cells, normal plasma cells, normal B cells with normal activation patterns and subset compositions, normal NK cells (Fig. 3d and Supplementary Data File S7). In conclusion, we did not identify any current comorbidities in subject 1.

### Generation of a *DNAH17*-specific phenotyping protocol

**Figure 4.**
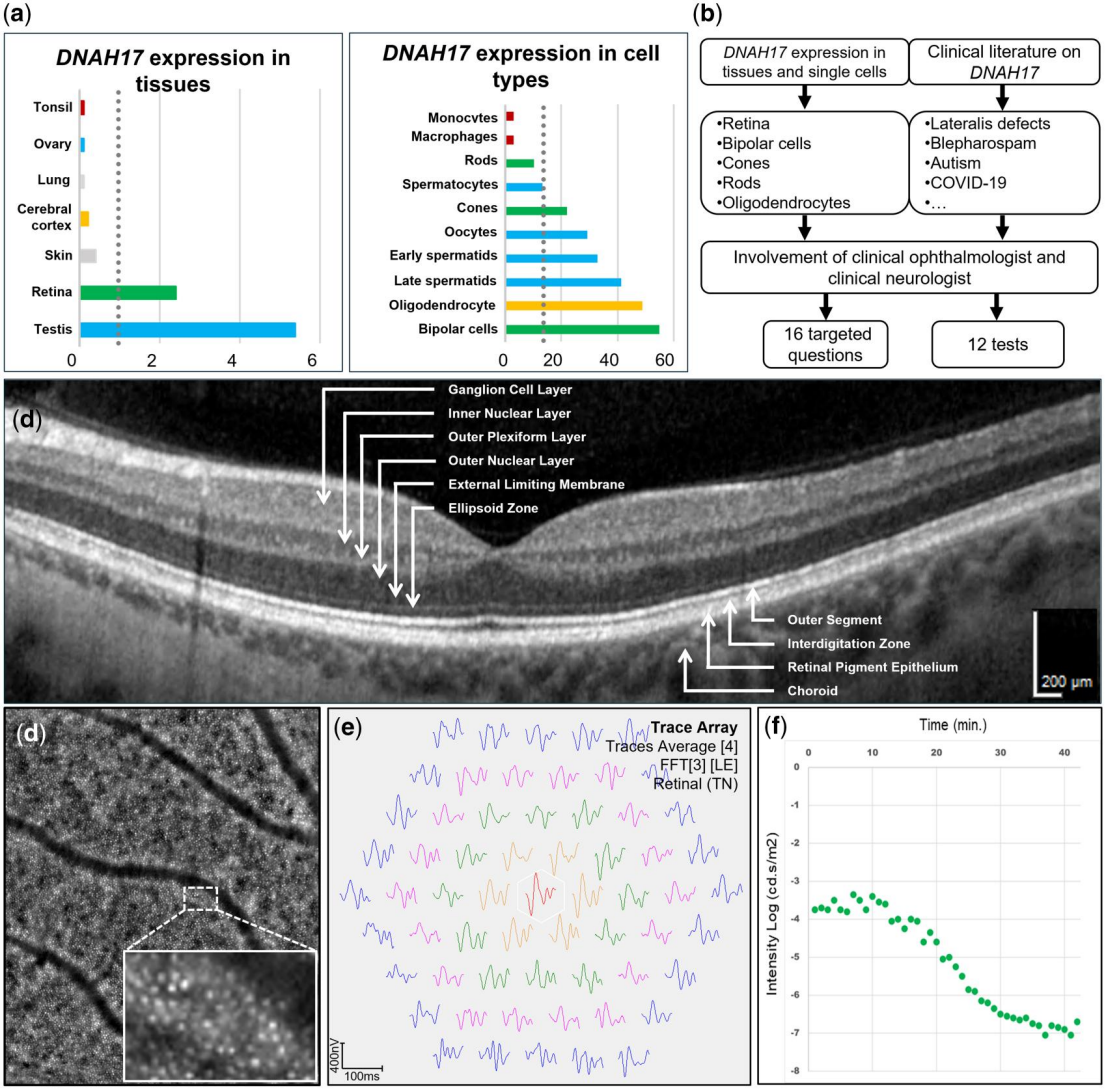
***DNAH17* expression, protocol development, and ophthalmological findings.** (**a**) Tissues (left) and single cell types (right) with highest relative expression of *DNAH17*. Gray-dotted line corresponds with 4-fold mean. Bar coloring: blue for gonadal tissue or cells; green for the retina or retinal cells, gray for other tissues, yellow for brain tissues or cells, red for immune tissues or cells. (**b**) Diagram summarizing the development of a DNAH17-specific phenotyping protocol. (**c**) An Optical Coherence Tomography (OCT) of the right eye reveals normal structural appearance of all retinal layers. On an OCT, photoreceptors span several layers: their cell bodies reside in the outer nuclear layer, their inner and outer segments extend toward the retinal pigment epithelium, and their synaptic terminals project into the outer plexiform layer. Here, they form synapses with bipolar cells, whose cell bodies are located in the inner nuclear layer. (**d**) Adaptive Optics imaging of the right eye, centered 4 degrees temporal of the fovea, reveals a normal and well-organized cone photoreceptor mosaic. (**e**) Multifocal electroretinography (mfERG) of the right eye shows normal responses of photoreceptor and bipolar cells across all retinal regions. Colored lines indicate the waveform responses at each stimulus location, while the colors represent different concentric retinal zones, from the central fovea outward to the peripheral retina. This allows for topographic analysis of retinal function. (**f**) Dark adaptation testing in both eyes shows normal rod and cone function, with visual sensitivity recovering at the expected rate. Green dots depict individual measurements of threshold sensitivity over time. The curve represents the expected recovery profile for healthy retinal function.

Subject 2 was referred to our fertility clinic for an unfulfilled child wish. Routine diagnostic work-up indicated an oligoasthenoteratozoospermia (Supplementary Data File S4). Genetic diagnostics showed a likely pathogenic variant and a VUS in the *DNAH17* gene (Supplementary Data File S4). The *DNAH17* gene encodes a dynein heavy chain protein that is part of the outer dynein arms of the sperm axoneme (Whitfield *et al.*, 2019; Sha *et al.*, 2020). It’s ATPase activity facilitates the beating of the flagella and *DNAH17* defects result in a reduced number of spermatozoa that have reduced motility and are frequently morphologically aberrant (Whitfield *et al.*, 2019; Sha *et al.*, 2020). As one of the parents was deceased, segregation analysis was no option, but long-read sequencing (30× high-fidelity [HiFi]) genome on a PacBio Revio system, performed as described previously (Höps *et al.*, 2025) showed that these variants were present in trans (compound heterozygous). The observed morphological abnormalities and motility characteristics of the spermatozoa as well as the frequent loss of the inner microtubule doublet of the sperms’ axoneme, as determined by electron microscopy (Supplementary Data File S4), resemble known spermatozoa features of *DNAH17* cases (Whitfield *et al.*, 2019; Tang *et al.*, 2025). This information allowed the VUS to be reclassified to a likely pathogenic variant. Altogether, we concluded that the *DNAH17* variants are likely the cause of subject’s 2 infertility. To determine if the gene showed expression outside the gonads, we assessed its expression pattern via the HPA portal. Abundant expression in the retina and several somatic cell types, including oligodendrocytes, was reported (Fig. 4a). From this, we concluded that the subject was suitable for inclusion in our study and subsequently approached him for participation. After registering his willingness to participate in this study, we again followed our seven-step protocol to develop a *DNAH17*-specific phenotyping protocol (Supplementary Data Files S5 and S6). The final version consists of 65 question/attentions points of which 16 are *DNAH17*-prompted. In addition, it also contains 12 tests to assess retina morphology and function (Supplementary Data File S8).

### Deep phenotyping results of *DNAH17* subject

Overall, the subject’s answers did not indicate any current medical issues (data not shown). The targeted questions addressing the presence of retinal or neuronal symptoms did not delineate potential comorbidities. Regarding the retina, this is in line with the test results. A normal morphology of the retinal layers, in which the photoreceptors and the bipolar cells reside, was observed (Fig. 4c and d; Supplementary Data File S8). Visualization of the cone photoreceptors revealed a normal and well-organized appearance (Fig. 4d). Further, retinal functionality regarding color vision and darkness adaptation was normal (Fig. 4e and f; Supplementary Data File S8). The physical examination revealed no indication of lateralis defects, dextrocardia, or neurological dysfunction. Follow-up investigations were therefore not performed. In conclusion, we did not identify any current comorbidities in subject 2.

## Discussion

In males, the link between reproductive fitness and general health has become strikingly apparent, especially for severe infertility (Aitken, 2025; Priskorn *et al.*, 2025). However, to derive clinical benefits from this correlation, further research is needed to decipher the exact relationship between infertility and general health. Reduction of reproductive fitness has various distinct causes, ranging from lifestyle and environmental factors to genetic factors or a combination of these (Agarwal *et al.*, 2021). Each specific cause could have its own specific effect on general health (Punjani and Lamb, 2020). In addition, these men may also experience unrelated health defects. Clearly, deciphering how the cause(s) of infertility affect general health will be complicated.

As a starting point, we therefore focused on the subpopulation of infertile males whose infertility stems from a single gene (monogenic) defect. Generally, for this group, the relationship between infertility and health seems to be more straightforward. This population is also growing in numbers with the introduction of panel-based genetic diagnostics for male infertility. Understanding whether these monogenic causes are associated with health implications beyond fertility, in the form of (potentially preventable) comorbidities, is therefore becoming increasingly relevant.

We present a framework to systematically screen these men for health issues. Central in our approach is the use of deep phenotyping as the main tool to discern comorbidities. We present a seven-step method for developing gene-specific deep phenotyping protocols that uses public gene-expression data and a literature screen for the causal gene. We have used our method to develop a gene-specific protocol for *MEI1*, a fertility gene that is also highly expressed in immune cells, and *DNAH17*, a fertility gene that is also highly expressed in retinal photoreceptor cells, bipolar cells, and oligodendrocytes. Central in the development of these protocols was an MMT that recurrently discussed the proposed questions and tests. This team weighted the burden for the participant versus the clinical relevance that a question or test yields. Since certain fertility genes are associated with increased risks of cancer (Punjani and Lamb, 2020; Wyrwoll and Steingröver, 2024) or highly expressed in an organ like the brain, such carefulness is called for. The screening of two men with *MEI1* and *DNAH17*-associated infertility did not reveal any current significant comorbidities. Additional screening of men with infertility due to pathogenic variants in these genes is needed to truly delineate comorbidities or lack thereof.

We acknowledge that our approach is in development and can be improved upon. For example, the transcription profiles we used from the Human Protein Atlas are derived from human adults. Potentially, comorbidities could arise prior to adulthood because of an abrogated developmental function of a fertility gene. We have chosen not to use developmental expression data because it is not easily accessible. This may change in the near future, allowing additional input for the specification of the deep phenotyping protocol. In the meantime, different aspects of fetal development and development during childhood are part of our standard questionnaire. Morbidity due to a gene’s developmental role might be identified by these questions. Another limitation is our restriction to tissue/cell types that have high gene expression levels in the Human Protein Atlas. Consequently, tissues/cell types with low expression levels are not represented in our protocol and potential comorbidities could be missed. Therefore, potentially existing comorbidities are missed. However, the alternative of taking more tissue/cell types in account has the danger of increasing the size of the analyses to an unworkable and unaffordable extend. In addition, we expect that general questions about medical history and the alertness of the clinician to identify health issues during the visit will help minimize the risk of missing comorbidities.

For many years, (sex) chromosome abnormalities were the sole identifiable cause for male infertility. The growing body of monogenetic causes is a relatively new development, and most identified variants are unique to a single patient or family. This poses a challenge to variant interpretation. Genetic variants may have variable clinical consequences, for example depending on residual gene function or genetic modifiers. This can be illustrated by the *DNAH17* gene. For this gene, more than 20 cases have been described and even more (likely) pathogenic genetic variants (Whitfield *et al.*, 2019; Song *et al.*, 2023; Muranishi *et al.*, 2024; Tang *et al.*, 2025). The range in residual activity of DNAH17 in these cases is likely responsible for the variety in progressive motile sperm in the semen of these men, which ranges from 0% to 17.5%. In addition, also phenotypic variance among individuals with the identical pathogenic variants can exist (Kingdom and Wright, 2022). Consequently, multiple subjects with variants in the same gene will need to be assessed by deep-phenotyping to accurately identify true genotype–phenotype associations. Moreover, a sufficiently large number of phenotyped men per gene is essential to distinguish genuine gene-specific comorbidities from unrelated ones and to determine the phenotypic variation of a co-morbidity within such a cohort. For example, subject 1 presented with recurrent ear infections during his childhood (data not shown). Whether *MEI1*-loss has a role in this, is unknown. Since recurrent ear infections are frequently occurring in the general population, only deep-phenotyping of multiple men with infertility due to MEI1-loss will clarify whether this condition is truly associated.

Because the number of recognized fertility-associated genes is approaching 150 (Riera-Escamilla *et al.*, 2025) (with a conservative estimate of ∼600 genes for azoospermia alone; Nagirnaja *et al.*, 2022) and the prevalence of a specific monogenic cause is low, the chance that a fertility clinician can perform multiple deep-phenotyping assessments for men with an identical genetic cause is low. Hence, the identification of morbidities associated with monogenic causes of infertility needs to be a concerted global effort. This effort would benefit from standardized questionnaires and underlaying principles, for which we present an initial framework. Future iterations of this framework by other groups will undoubtedly lead to its improvement. We envision a curated web-based environment where fertility specialists or clinical geneticists can upload their findings and explore comorbidities associated with specific genes. Our effort resulted in two gene-specific phenotyping protocols and, in a broader sense, phenotyping protocols for ‘adaptive immunological disorders’, ‘retinal pathologies’, and ‘axon-myelinating disorders’. These protocols can be used to facilitate deep phenotyping efforts by other groups.

The proposed framework is developed in a large university hospital. This setting allowed us to confer and work together with a wide range of departments to develop the gene-specific protocols. In addition, this setting was also beneficial for executing the protocol and taking medical tests in 1 day. However, such a setting is not a requirement to perform the deep phenotyping of a subject. Less extensive protocols, for example without extra medical tests, will also be insightful. The power for comorbidity identification will be in the number of assessments.

We applied our framework for gene-specific deep-phenotyping protocol development to infertile males. However, it can also be applied to women who are infertile due to a monogenic cause (Volozonoka *et al.*, 2022; Van Der Kelen *et al.*, 2023). The male and female germline share several important biological commonalities like primordial germ cell formation, primordial germ cell migration to the fetal gonads, and meiosis. Consequently, numerous male infertility genes are also (likely) female fertility genes. Indeed, *MEI1* is such a gene, associated with recurrent hydatidiform mole pregnancies, early embryonic arrest, and recurrent implantation failure in females (Dong *et al.*, 2021). In addition, there are also female-specific fertility genes (Volozonoka *et al.*, 2022; Van Der Kelen *et al.*, 2023). The investigation of the comorbidities associated with female fertility genes, is of equal urgency to that of the male counterparts.

Ultimately, this approach aims to provide clarity on the presence or absence of infertility-associated comorbidities on a gene-by-gene basis. This knowledge will enable clinicians to counsel patients on (possibly preventable) health risks and reproductive options. Consequently, medical care for individuals with infertility may, at times, extend beyond reproductive needs to general health.

## Supplementary Material

deag038_Supplementary_Data_File_S1

deag038_Supplementary_Data_File_S2

deag038_Supplementary_Data_File_S3

deag038_Supplementary_Data_File_S4

deag038_Supplementary_Data_File_S5

deag038_Supplementary_Data_File_S6

deag038_Supplementary_Data_File_S7

deag038_Supplementary_Data_File_S8

## Data Availability

Most of the data underlying this article is available in the article and in its online supplementary material. The only exception is the data that contains the answers of participants to the questionnaires. These cannot be publicly shared for the privacy of participants. This data will be shared on reasonable request to the corresponding author.
