# Supplementary material for: A clinical protocol for the detection of comorbidities associated with monogenic causes of male infertility
Source: Hum Reprod. 2026 Mar 21;41(5):689–98. doi: 10.1093/humrep/deag038 (PMC13139667; doi:10.1093/humrep/deag038)
Supplement: deag038_Supplementary_Data_File_S1 [file deag038_supplementary_data_file_s1.docx]

Supplementary Data File S1

**Proposed use of HPA portal**

Obtaining the tissues and cell types with fourfold increased expression from the HPA


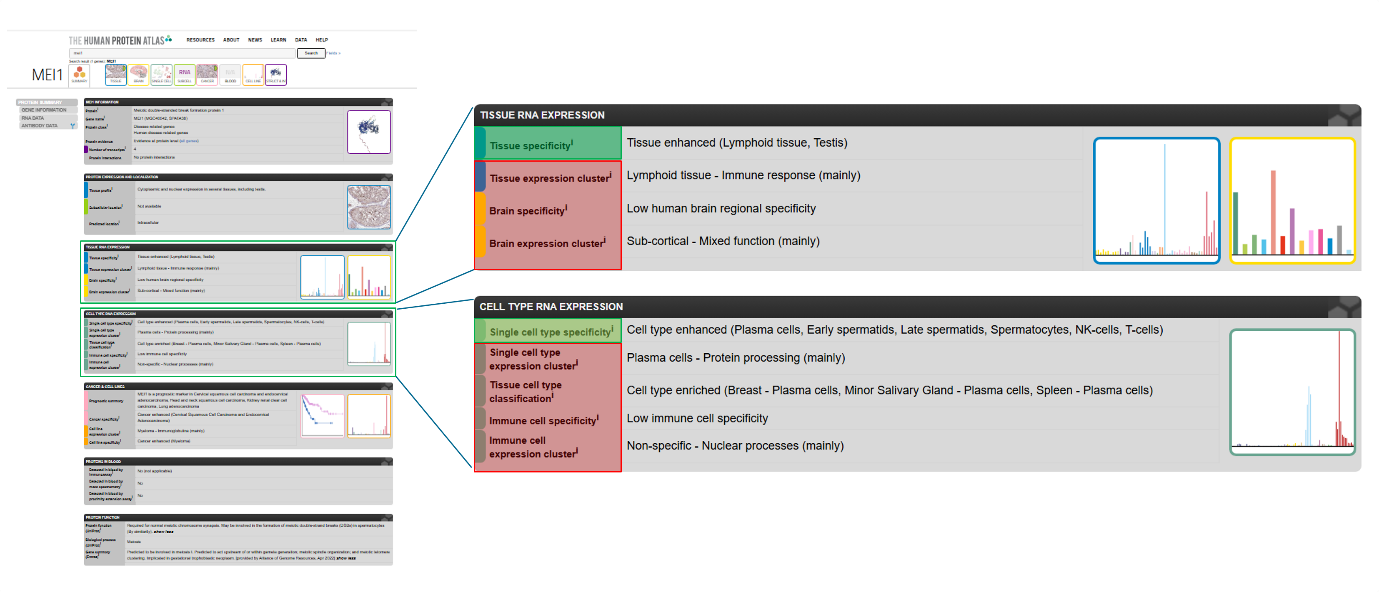


The HPA website has a data collation called *Protein Summary*. This page presents several summaries on different topics of a quarried gene. In the summary named *Tissue RNA Expression,* the tissues that have at least four times the mean value of all tissues for which expression was analyzed are listed under *Tissue specificity* (depicted in dark green)*.* In the summary *Cell Type RNA Expression*, the cell types that have at least four times the mean of all cell types for which expression was analyzed are listed under *Single Cell Type Specificity* (depicted in light green)*.* These listed tissues and cell types have been used as input for the gene-specific phenotyping protocol.

*23-5-2025*

*https://www.proteinatlas.org/ENSG00000167077-MEI1*
