# Supplementary material for: A clinical protocol for the detection of comorbidities associated with monogenic causes of male infertility
Source: Hum Reprod. 2026 Mar 21;41(5):689–98. doi: 10.1093/humrep/deag038 (PMC13139667; doi:10.1093/humrep/deag038)
Supplement: deag038_Supplementary_Data_File_S2 [file deag038_supplementary_data_file_s2.docx]

Supplementary Data File S2.

**Assessment of overlap between HPA and OMIM prompted tissues, organs and/or cell types for 17 genes causal for male syndromic infertility**

| **HGNC gene name** | **Disorder** | **OMIM number** | **Tissue listed in HPA under "Tissue specificity" for this gene^#^** | **Cell types listed in HPA under "Single cell type specificity" for this gene^#^** | **Organs and tissues selected for additional clinical attention based on HPA findings^#^** | **Organs, tissues or cell types with non-congenital co-morbidities reported in OMIM** | **Number OMIM-listed comorbidities for which HPA offers data in corresponding tissue/ cell type **** | **Tissues/cell types that overlap between OMIM and HPA for this gene** | **Number of overlapping targets** | **remarks** |
| --- | --- | --- | --- | --- | --- | --- | --- | --- | --- | --- |
| *APOA1* | Amyloidosis, heriditary systemic 2 | 105200 | liver | Hepatocytes, Proximal enterocytes | liver | liver, spleen, kidney, skin | 4 | liver | 1 |  |
| *CATSPER2* | Deafness-Infertility syndrome | 611102 | retina, testis | Rod photoreceptor cells, Cone photoreceptor cells, Early spermatids, Spermatocytes, Horizontal cells | eye | ear | 0** | none | 0 |  |
| *CCDC39* | Primary ciliary dyskinesia 14 | 613807 | lymphoid tissue, retina | Ciliated cells, Excitatory neurons, Early spermatids, Cone photoreceptor cells, Spermatocytes, Inhibitory neurons | eye, lung, brain | sinuses, ears, nose, lung | 1*^,^** | lung | 1 |  |
| *CCDC40* | Primary ciliary dyskinesia 15 | 613808 | choroid plexus, fallopian tube | Ciliated cells, Rod photoreceptor cells, Spermatocytes, Cone photoreceptor cells, Early spermatids | choroid plexus, lung, bronchus, eye | sinuses, ear, nose, lung | 1* | lung | 1 |  |
| *CDC14A* | Deafness, autosomal recessive 32 | 608653 | testis | Late spermatids, Astrocytes, Early spermatids | astrocytes | ear | 0** | none | 0 |  |
| *CEP290* | Leber Congenital Amaurosis | 611755 | None | Rod photoreceptor cells, Spermatocytes | eye | kidney, nasal epithelial cells, retina | 3 | eye | 1 |  |
| *DNAAF2* | Primary ciliary dyskinesia 10 | 612518 | None | None | none | middle ear, nose, airways, lung | 1* | none | 0 |  |
| *DNAAF4* | Primary ciliary dyskinesia 25 | 615482 | retina | Ciliated cells, Rod photoreceptor cells, Spermatocytes, Cone photoreceptor cells, Early spermatids | eye, lung, bronchus | middle ear, sinuses, lung | 1* | lung | 1 |  |
| *DNAAF6* | Primary ciliary dyskinesia 36 | 300991 | choroid plexus, fallopian tube, testis | Early spermatids, Ciliated cells | choroid plexus, lung, bronchus | sinuses, ear, lung | 1* | lung | 1 |  |
| *FANCA* | Occult Fanconi Anemia | NA (PS227650) | testis | Spermatocytes, Spermatogonia, Syncytiotrophoblasts, Oocytes | none | platelets, red blood cells, leukocytes | 3 | none | 0 |  |
| *LRRC6 (aka DNAAF11)* | Primary ciliary dyskinesia 19 | 614935 | choroid plexus, testis | Early spermatids, Ciliated cells, Spermatocytes, Excitatory neurons, Inhibitory neurons, Late spermatids | choroid plexus, central nervous system, lung, bronchus | sinuses, middle ear, nose, bronchus, lung | 1* | lung, bronchus | 1 |  |
| *MNS1* | NA | NA (PS258150) | choroid plexus, fallopian tube, testis | Early spermatids, Ciliated cells, Spermatocytes, Oocytes | choroid plexus, lung | mild respiratory symptoms | 1 | lung | 1 | Not reported in OMIM. Clinical data obtained from PMID: 30148830;31534215. |
| *NLRP3* | Muckle-Wells Syndrome | 191900 | bone marrow | monocytes | blood, immune system | ear, kidney, immune related issues of joints, skin, eye, mouth | 1*,** | immune system | 1 |  |
| *PKD1* | Polycystic kidney disease 1 | 173900 | brain | Excitatory neurons, Inhibitory neurons, Horizontal cells | central nervous system, eye | heart, vascular, liver, kidney, colon | 5 | none | 0 |  |
| *RSPH3* | Primary ciliary dyskinesia 32 | 616481 | Low tissue specificity | Early spermatids, Ciliated cells | bronchus, lung | ear, nose, lung | 1* | lung | 1 |  |
| *SPEF2* | Spermatogenic Failure 43 | 618751 | Low tissue specificity | Ciliated cells, Early spermatids, Excitatory neurons | bronchus, lung | middle ear, nose, bronchus, lung | 1* | lung, bronchus | 1 | Not reported in OMIM. Clinical data obtained from PMID: 31942643; 31278745; 31151990; 31048344 |
| *TRIM37* | Mulibrey Nanism | 253250 | testis | Early spermatids, Spermatocytes, Inhibitory neurons, Excitatory neurons, Late spermatids | central nervous system | heart, liver, brain, eye | 4 | brain | 1 |  |
| total number |  |  |  |  |  |  | 29 |  | 12 |  |

^#^Reproductive organs (testis) and germ cells (e.g. spermatocytes) are listed but disregarded for this assessment

*Morbidities with a shared underlaying cause are grouped as one. For example: in most primary ciliary dyskinesia syndromes infections in the nose, bronchus, lungs and ear stem from impaired ciliary clearance. Morbidities resulting from (auto) immune reactions were also grouped.

**The ear and its cell types are not included in HPA expression data sets. Related comorbidities of the ear were therefor disregarded in this assessment.
