# Supplementary material for: A clinical protocol for the detection of comorbidities associated with monogenic causes of male infertility
Source: Hum Reprod. 2026 Mar 21;41(5):689–98. doi: 10.1093/humrep/deag038 (PMC13139667; doi:10.1093/humrep/deag038)
Supplement: deag038_Supplementary_Data_File_S3 [file deag038_supplementary_data_file_s3.docx]

Supplementary Data File S3

**Assessment of Phenotyping Male Infertility Due to …………… Deficiency**

*Completed by:*

*Date:*

**Phenotyping Male Infertility**

- **Date of consultation**
- **Study ID**
- **Age**

**Medical History**

- **Past medical history**
- **Current medication use**
- **Review of organ systems**

**Head/Hair**

- Skin, hair, nails: any abnormalities?

**Ears**

- Hearing

**Eyes**

- Vision

**Nose**

- Anosmia (loss of smell)

**Mouth**

- Tooth development (missing elements/cavities)

**Cardiological**

- Congenital heart defect, cardiomyopathy, palpitations, imaging

**Pulmonary**

- Respiratory infections, lung problems

**Digestive Tract**

- Gastrointestinal issues

**Liver**

- Jaundice, liver problems

**Urogenital tract**

- Congenital anomalies of the kidney and urinary tract
- Kidney problems and blood pressure
- Congenital anomalies of the internal and external reproductive organs

**Extremities**

- Abnormalities/extra fingers or toes

**Neurological Tract**

- Epilepsy
- Movement problems (balance/coordination)
- Muscle tone

**Endocrine Functions**

- Hormones other than sex hormones examined

**Haematological System**

- Bruising, prolonged bleeding

**Skeletal Function**

- Hypermobility, growth abnormalities

**Skin**

- Eczema, hyper-/hypopigmentation

**Immune System**

**Social History**

- **Educational development**
- **Occupation**
- **Social status** (Note: do not assess presence of offspring)
  - Married, etc.
- **Involvement of social services or aids**
  - Assisted living? Extra support?

**Childhood History**

- **Birth history**
  - Pregnancy
  - Delivery
  - Gestational age
  - Birth weight
- **Congenital abnormalities**
- **Neonatal period**

**Developmental History**

- **Behavior**
- **Psychomotor development**
- **Speech & language development**
  - Were milestones achieved on time?

**Family History**

- **Pedigree of the family**
- **Siblings**
- **Father and his family**
- **Mother and her family**
- **Are parents consanguineous?**

**Physical Examination**

- **General appearance**
- **Length (SD)**
- **Weight (SD)**
- **Body Mass Index (BMI)**
- **Head circumference (SD)**
- **Arm span (span/length ratio)**
- **Facial appearance**
- **Thoracic**
- **Abdomen**
- **Spine**
- **Extremities**
- **Skin**
