# Supplementary material for: A clinical protocol for the detection of comorbidities associated with monogenic causes of male infertility
Source: Hum Reprod. 2026 Mar 21;41(5):689–98. doi: 10.1093/humrep/deag038 (PMC13139667; doi:10.1093/humrep/deag038)
Supplement: deag038_Supplementary_Data_File_S4 [file deag038_supplementary_data_file_s4.docx]

Supplementary Data File S4

**Andrological data, histology or sperm morphology and genetics for subject 1 and 2**

**Subject 1**

Table 1. General clinical parameters

| **Karyotype** | **Y deletions** | **Urological History** |
| --- | --- | --- |
| 46, XY | None | Orchidopexy left side at age 6 |

Table 2. Andrological parameters

| **Age (yrs)** | **Fertility Parameters** | | | | **Outcome semen analyses** |
| --- | --- | --- | --- | --- | --- |
|  | **FSH**  RV: (1.5-12 U/l) | **LH**  RV: (1.7-8.6 U/l) | **T**  RV: (10.5-37 nmol/l) | **TV**  Left-right |  |
| 34 | 2.3 | 4.1 | 14 | 15-15 | Azoospermia |

Table 3. Histological assessment of spermatogenesis

| **Testis Side** | **% ES** | **% RS** | **% SC** | **% SG** | **% SCO** | **% TS** | **Nr. of tubules assessed** |
| --- | --- | --- | --- | --- | --- | --- | --- |
| Right | 0 | 0 | 98 | 0 | 2 | 0 | 66 |

ES: Elongating Spermatids, RS: Round spermatids, SC: Spermatocytes; SG: Spermatogonia; SCO: Sertoli Cell Only; TS: Tubular Shadows

**b**

**a**

**
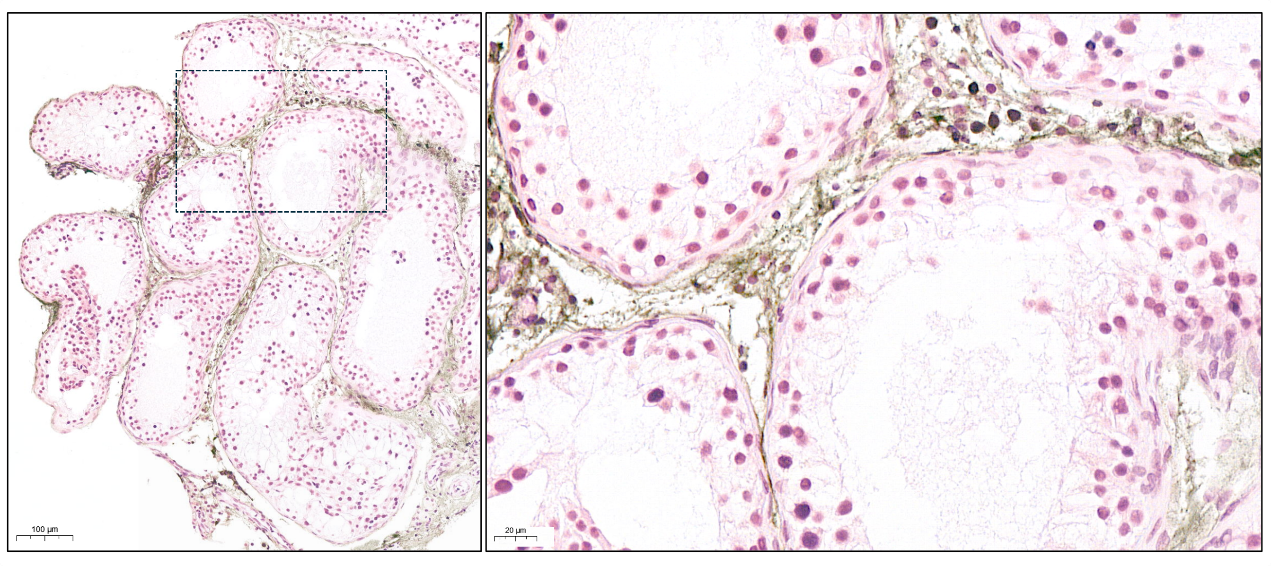
**

Figure 1. Testicular biopsy

**a-b)** Testis histology as determined via H&E staining. The indicated region in a, is shown enlarged in b. No spermatids were observed in the sections. Germ cells arrested at the meiotic stage.

Table 4. Assessment of genetic variants

| **Individual** | **Identified variants** | **MAF (gnomAD v4.1.0)** | **Classification According to ACMG-AMP Guidelines** |
| --- | --- | --- | --- |
| **Subject_1** | MEI1 Chr22(GRCh37):g.42172232C>T NM_152513.4:c.2671C>T p.(Gln891*); heterozygous, maternal | 0.000005577 (9/1613914) | Pathogenic (PVS1; PM2) |
|  | MEI1 Chr22(GRCh37):g.(42190597_42191311)_(42191964_42195111)del NM_152513.4: c.(3534+116_3535-104)_(3779+113_3780-147)del; heterozygous, paternal (deletion exon 29 and 30) | N.A. | Pathogenic (PVS1; PM2) |

**Subject 2**

Table 1. General clinical parameters

| **Karyotype** | **Y deletions** | **Urological History** |
| --- | --- | --- |
| 46, XY | None | None |

Table 2. Andrological parameters

| **Age (yrs)** | **Fertility Parameters** | | | | **Outcome semen analyses** |
| --- | --- | --- | --- | --- | --- |
|  | **FSH**  RV: (1.5-12 U/l) | **LH**  RV: (1.7-8.6 U/l) | **T**  RV: (10.5-37 nmol/l) | **TV**  (15-35 ml)  Left-right |  |
| 37 | 5.2 | 6.0 | 15 | 10-11 | Oligoasthenoteratozoospermia |

Table 3. Semen parameters

| **Analyses** | **Semen volume**  **(ml)** | **pH** | **Concentration (million/ml)** | **Total sperm number (million)** | **Motile (%)** | **Progressive motility (%)** | **Vitality (%)** |
| --- | --- | --- | --- | --- | --- | --- | --- |
| 1 | 2.3 | 7.5 | 2.5 | 5.8 | 16 | 8 | 90 |
| 2 | 2.6 | 7.7 | 2.8 | 7.3 | 17 | 9 | - |

77
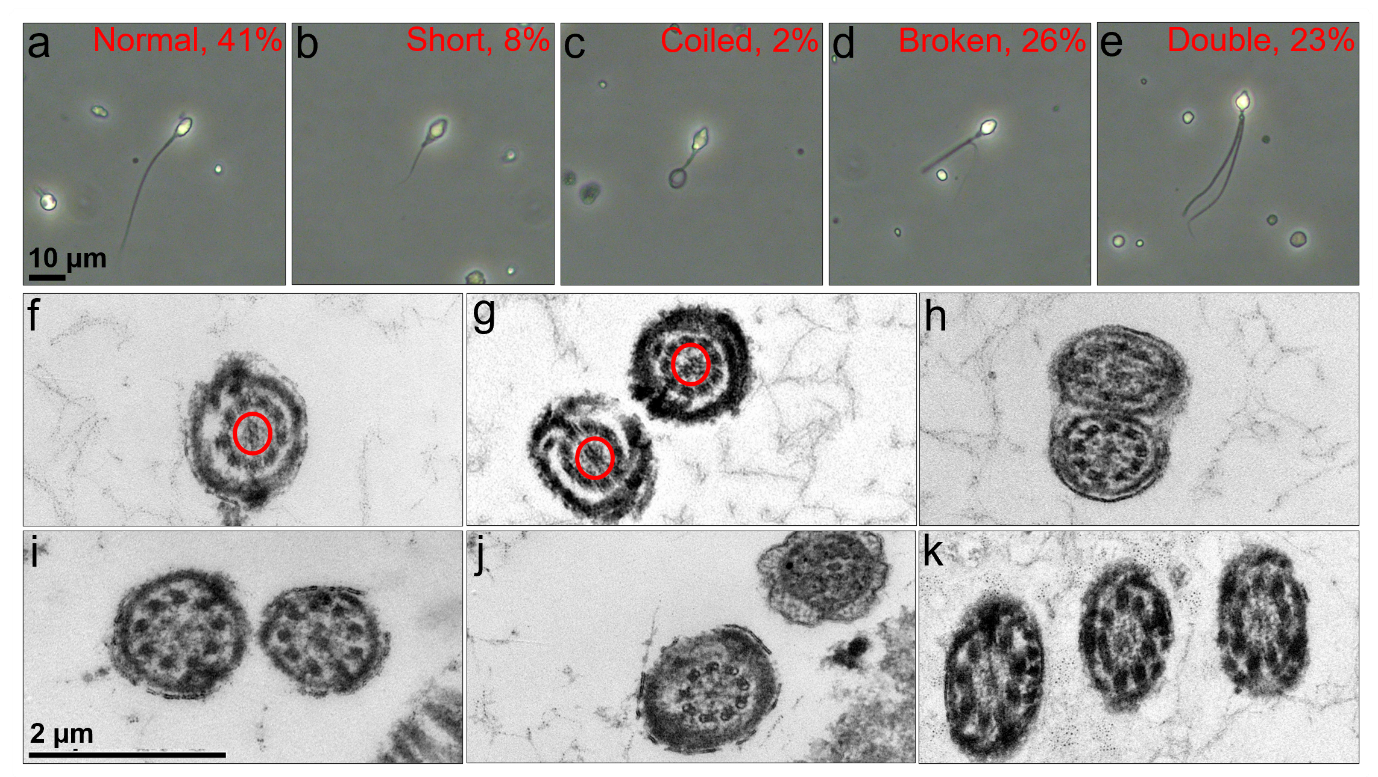


Figure 1. Flagellar morphological abnormalities of spermatozoa

**(a-e)** Examples of spermatozoa as found in the fresh ejaculate of subject 2. In red the percentage of spermatozoa observed with depicted morphology.

**(f-k)** Electron Microscopy images of spermatozoa of subject 2 with a focus on the axoneme. Axoneme structures with the typical 9+2 organization were observed (f and g). The inner doublet is marked with a red circle. The inner doublet was absent from numerous axonemes (h-k).

Table 4. Assessment of genetic variants

| **Individual** | **Identified variants** | **MAF (gnomAD v4.1.0)** | **Classification According to ACMG-AMP Guidelines** |
| --- | --- | --- | --- |
| **Subject_2** | DNAH17 Chr17(GRCh37):g.76471884G>A NM_173628.4:c.8186C>T p.(Pro2729Leu); heterozygous, paternal | N.A. | LP (PM2, PS3) |
|  | DNAH17 Chr17(GRCh37):g.76510927G>A NM_173628.4:c.4042C>T p.(Arg1348Cys); heterozygous, maternal | 0.004264 (6883/1614064) | LP (PM1; PM2, PS3) |
