# Supplementary material for: A clinical protocol for the detection of comorbidities associated with monogenic causes of male infertility
Source: Hum Reprod. 2026 Mar 21;41(5):689–98. doi: 10.1093/humrep/deag038 (PMC13139667; doi:10.1093/humrep/deag038)
Supplement: deag038_Supplementary_Data_File_S5 [file deag038_supplementary_data_file_s5.docx]

Supplementary Data File S5

**Development of *MEI1* and *DNAH17* specific deep phenotyping protocol in seven steps**

***MEI1***

**Step 1** By use of the HPA we identified the tissues and cell types with a four-fold increase in gene expression above the mean. These were: the spleen and the testis as tissues. Notably, expression in the bone marrow, an important immunological tissue, was just below the four-fold cut-off (figure 3a). Regarding cell types these were: plasma cells, early spermatids, late spermatids, spermatocytes, NK-cells and T-cells as cell types (see figure 3a, b). The expression of *MEI1* in the testis and germ cells is according to its described role in spermatogenesis (Ben Khelifa et al., 2018, Libby et al., 2002). Whereas the expression in immune tissues and cells, to our knowledge, has not been reported or studied. To gain more confidence in these observations we assessed the individual RNA bulk data sets that together make up the HPA meta data set. All show increased expression of *MEI1* in one or more lymphoid tissues, which strengthened our confidence that expression in immune cells was real.

**Step 2** Next, we assessed *MEI1* literature on clinically relevant findings. We combined the term “*MEI1*” with “human” or “clinical” for PubMed searches and identified 31 papers on male and female infertility and the role of *MEI1* in the formation of hydatidiform mole pregnancies (Dong et al., 2021). However, in the assessed literature we did not identify additional suggestions for morbidities (Supplementary Data File S6 ).

**Step 3** These findings were collated and shared with the MMT.

**Step 4** The MMT agreed that assessment of the functionality of the adaptive immune system of subject 1 should be the sole additional focus of the *MEI1*-specific phenotyping protocol. Since none of the MMT member was an expert on the adaptive immune system we identified and contacted a medical immunologist with this expertise.

**Step 5** The *MEI1* expression data was discussed with the medical immunologist. It was noted that expression was observed in cell types partaking in the humoral response as well as in cell types partaking in the cellular response. Specifically, the antibody producing plasma cells showed expression levels even higher than in germ cells (figure 3a). *MEI1* expression in the plasma cell progenitors, the B-cells, was just below the four-fold cut-off. T-cells and NK-cells, both important in the cellular response, showed expression levels above the four-fold cut off. Based on this we concluded that loss of *MEI1* could potentially affect both the humoral and cellular immune response. We therefore formulated questions aimed to identify dysfunctionality of the adaptive immune response for the participant. A total of 22 questions on various clinical symptoms associated with immunological disorders were formulated and incorporated in the general phenotyping protocol (Figure 3b, Supplementary Data File S7). Additionally, extensive phenotyping panels that are used in patient care to identify functionality of the humoral and cellular immune response were proposed in the preliminary *MEI1*-phenotyping protocol. These panels are used to determine concentrations of all immunoglobulin classes and the presence of auto-immune and monoclonal antibodies as well. A detailed immune cell phenotyping was proposed by quantification of various immune cell types as monocytes, neutrophils, T-cells, B-cells, and NK-cells and, in addition, a further characterization of different stages of T-cells and B-cells (including plasma cells) (Supplementary Data File S7 ).

**Step 6** The preliminary protocol was discussed with the MMT. All 22 questions were discussed and regarded as legitimate since they were aimed to identify morbidities. The burden for the patient of the drawing of blood needed for the immune cell evaluation was discussed. It was decided that the burden did not weigh up to the medical and scientific insight gained.

**Step 7** With all proposed questions and screenings approved the *MEI1*-specific phenotyping protocol was finalized (Supplementary Data File S7). This final version consists of 71 question/attentions points of which 22 are *MEI1*-prompted. In addition, it also contains six panels aimed to phenotype the adaptive immune response.

***DNAH17***

**Step 1** By use of the HPA portal, we identified the tissues and cell types with at least a four-fold increase in expression above the mean. Apart from the testis and the germ cells these were: the retina as tissue, and bipolar cells and photoreceptor cells as retinal cell types. Although its expression in different parts of the brain is not prominent, the cell-type data did indicate high expression of this gene in in oligodendrocytes (figure 4a).

**Step 2** We next searched PubMed for *DNAH17*-related literature. We combined the term “*DNAH17*” with “human” or “clinical” and retrieved a total of 52 papers. We scrutinized the retrieved papers for clinically relevant observations. In addition to male infertility, links between *DNAH17*-loss and laterality defects and dextrocardia (Breuer et al., 2022, Yu et al., 2022), blepharospasm (eyelid twitching) (Tian et al., 2018) and autism (Narita et al., 2020) were suggested.

**Step 3** These findings were collated and presented to the MMT.

**Step 4** The expression profile of *DNAH17* in the retina and retinal cells indicated a potential role of this gene in the visual system. To detect potential morbidities in these due to *DNAH17* deficiency we decided to ask the input from a clinical ophthalmologist. To delineate potential morbidities due to *DNAH17* deficiency in the oligodendrocytes it was decided to contact a clinical neurologist specialized in white matter pathology. Regarding our findings in the literature: the link with autism and blepharospasm was considered too weak to warrant targeted questions after critical assessment of the genetic data. Since blepharospasm is easily noticed it was added as an attention point to the physical examination part of the protocol. To diagnose laterality defects and dextrocardia, a chest ultrasound is most conclusive. However, this was deemed too intrusive for the participant. Therefore, we decided that these potential defects would first be assessed with acoustic palpitation and auscultation. If indicated, a follow-up ultrasound would be recommended to the participant. The *DNAH17* gene encodes a dynein heavy chain protein that is part of the outer dynein arms of the sperm axoneme. It’s ATPase activity facilitates the beating of the flagella and genetic *DNAH17* defects result in reduced number of spermatozoa that have reduced motility and are frequently morphological aberrant. Although the protein is found in the sperm flagella, it is absent from the respiratory flagella. This is reflected in the HPA expression data, which shows very low expression of *DNAH17* in the ciliated cell of the lung and bronchus

**Step 5** Expression and function of *DNAH17* in the retina were discussed with a clinical ophthalmologist. Several retinal pathologies are caused by disfunctions of cytoplasmic dynein motors, a class of proteins related to dynein heavy chain proteins (Dahl and Baehr, 2021). Also, dysfunction of the primary cilia in retinal cells causes impairment of the visual system (Chandra et al., 2022, Liu et al., 2024). Although the axoneme structure of the sperm flagellum is more complex than the primary cilia found in retinal cells, this does not exclude a detrimental effect of *DNAH17* deficiency in these retinal cells too. The retinal cell types with high expression either have a function in converting light into biological signals (cone photoreceptor cells and rod photoreceptor cells) or in relaying signals from these photoreceptor cells (bipolar cells). Altogether, we therefore reasoned that impaired visual function could represent a *DNAH17*-associated comorbidity. In addition, since *DNAH17* deficiency results in malformation of the spermatozoa, we hypothesized that the morphological appearance of the retina at a cellular level could also be affected. We therefore formulated questions and identified tests that could indicate impairments in both of retinal function and morphology. Specifically, basic measurements such as refraction, visual acuity testing, and color vision testing using HRR were proposed to be supplemented with a fundoscopy, fundus imaging, optical coherence tomography and adaptive optics to determine and evaluate the morphology of the retinal layers, in which the photoreceptors and bipolar cells reside. In addition, an electroretinogram and a dark adaptation test were proposed to assess retinal function.

Another cell type with high *DNAH17* expression are oligodendrocytes (figure 4a). These are the axon-myelinating cells in the central nervous system. In contrast to the retinal cells, no link between oligodendrocytes and cilia function has been reported. Dysfunction of oligodendrocytes generally leads to hypomyelinating leukodystrophies (Wolf et al., 2021). Common signs include nystagmus (uncontrolled movements of the eye), mildly delayed development, learning difficulties and balance problems. To diagnose a leukodystrophy, a thorough medical history with full attention for these signs, a neurological assessment, and Magnetic Resonance Imaging (MRI) of the brain were proposed.

In total, 17 targeted questions, 12 tests/measurements for retinal morphology and functionality, and two oligodendrocyte-related tests were proposed.

**Step 6** The MMT discussed the proposed tests. Since none of the retinal experiments were invasive and all provided relevant information about the retinal condition, they were approved. The use of an eye-sedative and pupil-dilating eye drops, required for some experiments, was not considered a problem, provided that the participant was informed and gave consent. All proposed examinations are standard procedures in clinical care. When the proposal to diagnose impairment due to oligodendrocyte was discussed the burden for the participant versus the scientific and medical gain were at odds according to the MMT. While the targeted medical history was approved, the neurological assessment and MRI were not. Both were considered too burdensome for the participant to apply at first instance. If the targeted medical history yielded suggestions of neurological pathology the neurological assessment and MRI would be discussed with the participant. Specifically for the MRI the chance of incidental findings was discussed. Whilst a finding in the context of research is very undesirable and should be avoided, an MRI is currently the superior method to detect abnormalities of the brain’s white matter. A second option was discussed in which the MRI operator would be asked to limit the observations strictly on the appearance of the white matter. In the end the MMT chose to perform the MRI only on indication, because this minimized the burden for the subject.

**Step 7** The approved questions and tests were incorporated, and the *DNAH17*-specific phenotyping protocol was finalized (Supplementary Data File S8). The final version consists of 65 question/attentions points of which 16 are *DNAH17*-prompted. In addition, it also contains 12 tests to assess retina morphology and function.

Ben Khelifa M, Ghieh F, Boudjenah R, Hue C, Fauvert D, Dard R, Garchon HJ, Vialard F. A MEI1 homozygous missense mutation associated with meiotic arrest in a consanguineous family. *Hum Reprod* 2018;33: 1034-1037.

Breuer K, Riedhammer KM, Müller N, Schaidinger B, Dombrowsky G, Dittrich S, Zeidler S, Bauer UMM, Westphal DS, Meitinger T *et al.* Exome sequencing in individuals with cardiovascular laterality defects identifies potential candidate genes. *Eur J Hum Genet* 2022;30: 946-954.

Chandra B, Tung ML, Hsu Y, Scheetz T, Sheffield VC. Retinal ciliopathies through the lens of Bardet-Biedl Syndrome: Past, present and future. *Prog Retin Eye Res* 2022;89: 101035.

Dahl TM, Baehr W. Review: Cytoplasmic dynein motors in photoreceptors. *Mol Vis* 2021;27: 506-517.

Dong J, Zhang H, Mao X, Zhu J, Li D, Fu J, Hu J, Wu L, Chen B, Sun Y *et al.* Novel biallelic mutations in MEI1: expanding the phenotypic spectrum to human embryonic arrest and recurrent implantation failure. *Hum Reprod* 2021;36: 2371-2381.

Libby BJ, De La Fuente R, O'Brien MJ, Wigglesworth K, Cobb J, Inselman A, Eaker S, Handel MA, Eppig JJ, Schimenti JC. The mouse meiotic mutation mei1 disrupts chromosome synapsis with sexually dimorphic consequences for meiotic progression. *Dev Biol* 2002;242: 174-187.

Liu X, Pacwa A, Bresciani G, Swierczynska M, Dorecka M, Smedowski A. Retinal primary cilia and their dysfunction in retinal neurodegenerative diseases: beyond ciliopathies. *Mol Med* 2024;30: 109.

Narita A, Nagai M, Mizuno S, Ogishima S, Tamiya G, Ueki M, Sakurai R, Makino S, Obara T, Ishikuro M *et al.* Clustering by phenotype and genome-wide association study in autism. *Transl Psychiatry* 2020;10: 290.

Tian J, Vemula SR, Xiao J, Valente EM, Defazio G, Petrucci S, Gigante AF, Rudzińska-Bar M, Wszolek ZK, Kennelly KD *et al.* Whole-exome sequencing for variant discovery in blepharospasm. *Mol Genet Genomic Med* 2018;6: 601-626.

Wolf NI, Ffrench-Constant C, van der Knaap MS. Hypomyelinating leukodystrophies - unravelling myelin biology. *Nat Rev Neurol* 2021;17: 88-103.

Yu X, Yuan L, Deng S, Xia H, Tu X, Deng X, Huang X, Cao X, Deng H. Identification of DNAH17 Variants in Han-Chinese Patients With Left-Right Asymmetry Disorders. *Front Genet* 2022;13: 862292.
