# Supplementary material for: A clinical protocol for the detection of comorbidities associated with monogenic causes of male infertility
Source: Hum Reprod. 2026 Mar 21;41(5):689–98. doi: 10.1093/humrep/deag038 (PMC13139667; doi:10.1093/humrep/deag038)
Supplement: deag038_Supplementary_Data_File_S6 [file deag038_supplementary_data_file_s6.docx]

Supplementary Data File S6

**MEI1 and DNAH17 literature searches**

**MEI1**

Search terms: “MEI1 and human”, “MEI1 and clinical”.

Yield: 15 papers.

All papers (4) not related to spermatogenesis or infertility are shown below and accompanied with i) a brief description of the main finding, ii) whether the paper resulted in amendment of the phenotyping protocol and iii) the argumentation for this.

| 1. | [A predictive study of metabolism reprogramming in cervical carcinoma.](https://pubmed.ncbi.nlm.nih.gov/35530966/)  Dai G, Ou J, Wu B.  Ann Transl Med. 2022 Apr;10(7):414. doi: 10.21037/atm-22-981.  PMID: 35530966  **Comments**  MEI1 related finding: MEI1 is part of a group of genes whose expression level is presented as a possible indicator of prognosis for invasive cervical carcinoma.  Did this paper result in additional targeted questions/clinical tests in the final protocol: No.  Why: Studied disease context not relevant. |
| --- | --- |
| 2. | [Human papillomavirus elevated genetic biomarker signature by statistical algorithm.](https://pubmed.ncbi.nlm.nih.gov/32537823/)  Tripathi N, Keshari S, Shahi P, Maurya P, Bhattacharjee A, Gupta K, Talole S, Kumar M.  J Cell Physiol. 2020 Dec;235(12):9922-9932. doi: 10.1002/jcp.29807.  PMID: 32537823  **Comments**  MEI1 related finding: MEI1 is part of a group of genes that are overexpressed in head and neck squamous cell carcinoma cases.  Did this paper result in additional targeted questions/clinical tests in the final protocol: No  Why: Different direction of expression. Studied disease context not relevant. |
| 3. | [Genomic characterization of cervical cancer based on human papillomavirus status.](https://pubmed.ncbi.nlm.nih.gov/30581036/)  Zhang L, Jiang Y, Lu X, Zhao H, Chen C, Wang Y, Hu W, Zhu Y, Yan H, Yan F.  Gynecol Oncol. 2019 Mar;152(3):629-637. doi: 10.1016/j.ygyno.2018.12.017. Epub 2018 Dec 20.  PMID: 30581036  **Comments**  MEI1 related finding: In cervical tumors, MEI1 is part of a group of genes whose expression differs in HPV negative tumors from HPV positive tumors.  Did this paper result in additional targeted questions/clinical tests in the final protocol: No  Why: Different direction of expression. Studied disease context not relevant. |
| 4. | [Gene-Expression Profiles in Generalized Aggressive Periodontitis: A Gene Network-Based Microarray Analysis.](https://pubmed.ncbi.nlm.nih.gov/26136121/)  Guzeldemir-Akcakanat E, Sunnetci-Akkoyunlu D, Orucguney B, Cine N, Kan B, Yılmaz EB, Gümüşlü E, Savli H.  J Periodontol. 2016 Jan;87(1):58-65. doi: 10.1902/jop.2015.150175. PMID: 26136121 |

**Comments**

MEI1 related finding: MEI1 is part of a group of genes that shows increased levels of expression in tissue biopsies from people with generalized aggressive periodontitis.

Did this paper result in additional targeted questions/clinical tests in the final protocol: No

Why: Different direction of expression.

**DNAH17**

Search terms: “DNAH17 and human”, “DNAH17 and clinical”.

Yield: 42 papers.

Reports on the long non-coding RNA named *DNAH17-AS1* were disregarded. All papers (15) not related to spermatogenesis or infertility are shown below and accompanied with i) a brief description of the main finding, ii) whether the paper resulted in amendment of the phenotyping protocol and iii) the argumentation for this.

| 1. | [Identification of Novel Genomic Variants in COVID-19 Patients Using Whole-Exome Sequencing: Exploring the Plausible Targets of Functional Genomics.](https://pubmed.ncbi.nlm.nih.gov/39557769/)  Mir R, Altemani FH, Algehainy NA, Alanazi MA, Elfaki I, Alsayed BA, Mir MM, Mustafa SK, Moawadh MS, Tayeb FJ, Alfaifi J, Alatawi SM, Alhiwety MS, Ullah MF.  Biochem Genet. 2024 Nov 18. doi: 10.1007/s10528-024-10970-8. PMID: 39557769  DNAH17 related finding: DNAH17 is part of a group of genes in which specific SNPs are associated with a decreased COVID-19 risk.  Did this paper result in additional targeted questions/clinical tests in the final protocol: No.  Why: Small effect of finding. Episodes of respiratory illness are part of the phenotyping questionnaire already. |
| --- | --- |
| 2. | [Unraveling the protective genetic architecture of COVID-19 in the Brazilian Amazon.](https://pubmed.ncbi.nlm.nih.gov/39521879/)  Barros MC, de Souza JES, Gomes DHF, Pinho CT, Silva CS, Braga-da-Silva C, Cavalcante GC, Magalhães L, Azevedo-Pinheiro J, Quaresma JAS, Falcão LFM, Costa PF, Salgado CG, Carneiro TX, Burbano RR, Dos Santos Vieira JR, Santos S, Soares-Souza GB, de Souza SJ, Ribeiro-Dos-Santos Â.  Sci Rep. 2024 Nov 9;14(1):27332. doi: 10.1038/s41598-024-78170-3.  PMID: 39521879.  DNAH17 related finding: DNAH17 is part of a group of genes in which specific SNPs are associated with a decreased COVID-19 risk.  Did this paper result in additional targeted questions/clinical tests in the final protocol: No.  Why: Episodes of respiratory illness are part of the phenotyping questionnaire already. |
|  |  |
| 3. | [Genetic change investigation in DOCK1 gene in an Iranian family with sign and symptoms of temporomandibular joint disorder (TMD).](https://pubmed.ncbi.nlm.nih.gov/39020145/)  Najafi S, Hashemi-Gorji F, Roudgari H, Goudarzi M, Jafarzadegan AM, Sheykhbahaei N.  Clin Oral Investig. 2024 Jul 18;28(8):432. doi: 10.1007/s00784-024-05819-8.  PMID: 39020145  DNAH17 related finding: DNAH17 is among a group of genes in which SNVs are found in the DNA of four TMD patients.  Did this paper result in additional targeted questions/clinical tests in the final protocol: No.  Why: The authors of the paper do not consider the SNV in DNAH17 as a plausible candidate. |
|  |  |
| 4. | [Designing of neoepitopes based vaccine against breast cancer using integrated immuno and bioinformatics approach.](https://pubmed.ncbi.nlm.nih.gov/37584493/)  Shuaib M, Singh AK, Gupta S, Alasmari AF, Alqahtani F, Kumar S.  J Biomol Struct Dyn. 2024 Oct;42(16):8624-8637. doi: 10.1080/07391102.2023.2247081. Epub 2023 Aug 16.  PMID: 37584493  DNAH17 related finding: DNAH17 is part of a group of proteins that are found to be frequently mutated in a breast invasive carcinoma data set. Based on this it is used as a template for neoepitope prediction.  Did this paper result in additional targeted questions/clinical tests in the final protocol:  Why: Findings do not relate to potential clinical symptoms of DNAH17 loss. |
| 5. | [Whole-exome sequencing study of hypospadias.](https://pubmed.ncbi.nlm.nih.gov/37168556/)  Chen Z, Lei Y, Finnell RH, Ding Y, Su Z, Wang Y, Xie H, Chen F.  iScience. 2023 Apr 12;26(5):106663. doi: 10.1016/j.isci.2023.106663. eCollection 2023 May 19.  PMID: 37168556  DNAH17 related finding: Genes encoding outer dynein arm heavy chain proteins, of which DNAH17 is one, are claimed to be enriched for mutations in a cohort of men with hypospadias.  Did this paper result in additional targeted questions/clinical tests in the final protocol: No.  Why: Presence of hypospadias is part of the medical urological history which is taken by the urologist. |
| 6. | [Identification of *DNAH17* Variants in Han-Chinese Patients With Left-Right Asymmetry Disorders.](https://pubmed.ncbi.nlm.nih.gov/35692830/)  Yu X, Yuan L, Deng S, Xia H, Tu X, Deng X, Huang X, Cao X, Deng H.  Front Genet. 2022 May 27;13:862292. doi: 10.3389/fgene.2022.862292. PMID: 35692830  DNAH17 related finding: A possible association between DNAH17 gene variants and left–right asymmetry disorders. Thirteen subjects carrying DNAH17 variants were studied. Two of these showed left–right asymmetry disorders, six did not and five 5 it remained unknown.  Did this paper result in additional targeted questions/clinical tests in the final protocol: Yes.  Why: Although based on these data a true association between left–right asymmetry disorders and DNAH17 loss is still uncertain, we decided that checking for indications for a left–right asymmetry disorder during the physical exam adds no burden to the participant. |
| 7. | [Exome sequencing in individuals with cardiovascular laterality defects identifies potential candidate genes.](https://pubmed.ncbi.nlm.nih.gov/35474353/)  Breuer K, Riedhammer KM, Müller N, Schaidinger B, Dombrowsky G, Dittrich S, Zeidler S, Bauer UMM, Westphal DS, Meitinger T, Dakal TC, Hitz MP, Breuer J, Reutter H, Hilger AC, Hoefele J.  Eur J Hum Genet. 2022 Aug;30(8):946-954. doi: 10.1038/s41431-022-01100-2. Epub 2022 Apr 26.  PMID: 35474353  DNAH17 related finding: DNAH17 is part of a group of genes in which genetic variants were identified in patients with cardiovascular laterality defects.  Did this paper result in additional targeted questions/clinical tests in the final protocol: No.  Why: The authors identified no additional homozygous or compound heterozygous rare variants in DNAH17 when they screened exomes of 2109 individuals with situs inversus totalis, heterotaxy or isolated CHD. Therefore, we considered the evidence too weak to burden the patient. |
| 8. | [Whole-exome sequencing identifies biosignatures that predict adverse survival outcomes in surgically treated patients with oral cavity squamous cell carcinoma.](https://pubmed.ncbi.nlm.nih.gov/34700279/)  Liao CT, Yang LY, Lee LY, Lin CY, Wang HM, Ng SH, Yen TC, Fan WL, Hsieh JC.  Oral Oncol. 2021 Nov;122:105547. doi: 10.1016/j.oraloncology.2021.105547. Epub 2021 Oct 23.  PMID: 34700279 |
|  | DNAH17 related finding: DNAH17 is claimed to be among a group of genes that allow prediction of survival of surgically treated patients with oral cavity squamous cell carcinoma.  Did this paper result in additional targeted questions/clinical tests in the final protocol: No.  Why: Findings do not relate to potential clinical symptoms of DNAH17-loss. |
| 9. | [Clustering by phenotype and genome-wide association study in autism.](https://pubmed.ncbi.nlm.nih.gov/32807774/)  Narita A, Nagai M, Mizuno S, Ogishima S, Tamiya G, Ueki M, Sakurai R, Makino S, Obara T, Ishikuro M, Yamanaka C, Matsubara H, Kuniyoshi Y, Murakami K, Ueno F, Noda A, Kobayashi T, Kobayashi M, Usuzaki T, Ohseto H, Hozawa A, Kikuya M, Metoki H, Kure S, Kuriyama S.  Transl Psychiatry. 2020 Aug 17;10(1):290. doi: 10.1038/s41398-020-00951-x.  PMID: 32807774 |
|  | DNAH17 related finding: GWAS of autism spectrum disorder identifies a group of loci, among others intronic DNAH17 ones.  Did this paper result in additional targeted questions/clinical tests in the final protocol: No.  Why: This result was not found in a replication cohort. Level of evidence does not credit further investigation. |
| 10. | [Undifferentiated embryonal sarcoma of the liver in a child: A whole exome sequencing analysis.](https://pubmed.ncbi.nlm.nih.gov/28539230/)  Kim JH, Sio CA, Park H, Kim H, Shin HD, Jung K.  Dig Liver Dis. 2017 Aug;49(8):944-946. doi: 10.1016/j.dld.2017.04.020. Epub 2017 May 3.  PMID: 28539230. |
|  | DNAH17 related finding: DNAH17 is part of a group of genes in which mutations are found in an embryonal sarcoma of a patient.  Did this paper result in additional targeted questions/clinical tests in the final protocol: No.  Why: No further similar findings reported since 2017. Genetic association too weak to burden the patient. |
|  |  |
| 11. | [Impact of Genomic Alterations on the Clinical Outcome of Patients with Hepatitis B-related Hepatocellular Carcinoma Receiving Curative Surgery: A Retrospective Cohort Study.](https://pubmed.ncbi.nlm.nih.gov/37772577/)  Wang CC, Fan WL, Liu TT, Pang ST, Wang JH, Huang CC, Huang EY, Chen YH.  Anticancer Res. 2023 Oct;43(10):4709-4722. doi: 10.21873/anticanres.16667.  PMID: 37772577  DNAH17 related finding: DNAH17 is among the top 10 most frequently mutated genes in in a cohort of patients with hepatitis B-related hepatocellular carcinoma.  Did this paper result in additional targeted questions/clinical tests in the final protocol: No.  Why: Liver function is part of the phenotyping questionnaire. Evidence too weak to warrant additional tests. |
| 12. | [Senescence-associated secretory phenotype constructed detrimental and beneficial subtypes and prognostic index for prostate cancer patients undergoing radical prostatectomy.](https://pubmed.ncbi.nlm.nih.gov/37624511/)  Feng D, Wang J, Li D, Wu R, Wei W, Zhang C.  Discov Oncol. 2023 Aug 25;14(1):155. doi: 10.1007/s12672-023-00777-1.  PMID: 37624511  DNAH17 related finding: DNAH17 is claimed to be among a group of genes with highest mutations rates for detrimental and beneficial senescence-associated secretory phenotype in prostate cancer.  Did this paper result in additional targeted questions/clinical tests in the final protocol: No.  Why: Findings do not relate to potential clinical symptoms of DNAH17-loss. |
| 13. | [Identification of Potential Biological Factors Affecting the Treatment of Ticagrelor After Percutaneous Coronary Intervention in the Chinese Population.](https://pubmed.ncbi.nlm.nih.gov/35082514/)  Yuan D, Shi X, Gao L, Wan G, Zhang H, Yang Y, Zhao Y, Sun D.  Pharmgenomics Pers Med. 2022 Jan 20;15:29-43. doi: 10.2147/PGPM.S338287. eCollection 2022.  PMID: 35082514  DNAH17 related finding: DNAH17 is in a group of genes that are associated with the expected antiplatelet effect of Ticagrelor, the receptor antagonist of P2Y12.  Did this paper result in additional targeted questions/clinical tests in the final protocol: No.  Why: Findings do not relate to potential clinical symptoms of DNAH17-loss. |
| 14. | [Genomic Alteration Characterization in Colorectal Cancer Identifies a Prognostic and Metastasis Biomarker: FAM83A\|IDO1.](https://pubmed.ncbi.nlm.nih.gov/33959500/)  Liu Z, Zhang Y, Dang Q, Wu K, Jiao D, Li Z, Sun Z, Han X.  Front Oncol. 2021 Apr 20;11:632430. doi: 10.3389/fonc.2021.632430. eCollection 2021.  PMID: 33959500.  DNAH17 related finding: The co-occurrence of DNAH17-MDN1 mutations is reported in aggregated genetic data of patients with colorectal cancer.  Did this paper result in additional targeted questions/clinical tests in the final protocol: No.  Why: Topic does not relate to potential clinical symptoms of DNAH17-loss. |
| 15. | [Whole-exome sequencing for variant discovery in blepharospasm.](https://pubmed.ncbi.nlm.nih.gov/29770609/)  Tian J, Vemula SR, Xiao J, Valente EM, Defazio G, Petrucci S, Gigante AF, Rudzińska-Bar M, Wszolek ZK, Kennelly KD, Uitti RJ, van Gerpen JA, Hedera P, Trimble EJ, LeDoux MS.  Mol Genet Genomic Med. 2018 May 16;6(4):601-26. doi: 10.1002/mgg3.411.  PMID: 29770609  DNAH17 related finding: A cohort of subjects with blepharospasm (involuntary eyelid movements) were genetically analyzed. DNAH17 is part of a group of genes in which deleterious variants are found.  Did this paper result in additional targeted questions/clinical tests in the final protocol: No. If present, involuntary eyelid movements will be noted by the clinical geneticist who performs the phenotyping.  Why: The genetic evidence for an association is relatively weak: present in a heterozygous state while being a known recessive gene. In addition, blepharospasm is very noticeable when present. His precludes the need for questioning. |
