# Supplementary material for: A clinical protocol for the detection of comorbidities associated with monogenic causes of male infertility
Source: Hum Reprod. 2026 Mar 21;41(5):689–98. doi: 10.1093/humrep/deag038 (PMC13139667; doi:10.1093/humrep/deag038)
Supplement: deag038_Supplementary_Data_File_S7 [file deag038_supplementary_data_file_s7.docx]

Supplementary Data File S7

**MEI1 phenotyping protocol and extended description of MEI1 immune phenotyping**

**Phenotyping Male Infertility**

- **Date of consultation:**
- **Study ID:**
- **Age:**

**Medical History**

- **Past medical history**
- **Current medication use**

**Review of Organ Systems**

**Head/hair**Skin, hair, nails: any abnormalities?

**Ears**

- Hearing

**Eyes**

- - - Vision

**Nose**

- Anosmia (loss of smell)

**Mouth**

- Tooth development (missing elements/cavities)

**Cardiological**

- Congenital heart defect, cardiomyopathy, palpitations, imaging

**Pulmonary**

- - - Respiratory infections, lung problems

**Digestive tract**

- - - Gastrointestinal issues
    - **MEI1-specific**: Do you suffer from chronic diarrhea?

**Liver**

- - - Jaundice, liver problems

**Urogenital tract**

- - - Congenital anomalies of the kidney and urinary tract
    - Kidney problems and blood pressure
    - Congenital anomalies of the internal and external reproductive organs

**Extremities**

- - - Abnormalities/extra fingers or toes

**Neurological tract**

- - - Epilepsy
    - Movement problems (balance/coordination)
    - Muscle tone
    - **MEI1-specific**:
      - Do you have problems with balance or coordination?

**Endocrine functions**

- - - Hormones other than sex hormones examined

**Hematological system**

- Bruising, prolonged bleeding

**Skeletal function**

- Hypermobility, growth abnormalities

**Skin**

- - Eczema, hyper-/hypopigmentation
  - **MEI1-specific**:
    - Are you hypersensitive to sunlight?
    - Do you have vitiligo (depigmentation of the skin)?
    - As a child, were you sensitive to sunlight / did you suffer from eczema / vitiligo?
    - Do you have partial albinism?
    - Do you have extensive warts?

**Immune System**

- **MEI1-specific**:
  - Are you known to have an autoimmune disease?
  - Do you frequently have bacterial infections (estimate number)?
  - How does your body respond to infections? Do you get a fever?
  - Do you ever suffer from swollen or painful lymph nodes?
  - Do you have asthma/COPD? Is it difficult to manage and steroid-dependent?
  - Do you suffer from unexplained bronchiectasis?
  - Do you frequently have upper respiratory tract infections?
  - Have your tonsils been removed?
  - Do you have recurrent severe infections (e.g., meningitis, osteomyelitis, arthritis)?
  - Are you known to have infections with atypical presentation, severe or unusually chronic course, unexpected pathogens, or opportunistic microorganisms?
  - Are you known to have vaccination complications? (e.g., BCG-itis, vaccinia generalisata)?
  - Do you suffer from malignancies (especially lymphomas)?

**Social History**

- **Educational development**
- **Social status**  (Note: do not assess presence of offspring)
  - Married, etc.: Registered partner
- **Involvement of social services or aids**
  - Assisted living? Extra support?

**Childhood History**

- **Birth history**
  - Pregnancy
  - Delivery
  - Gestational age
  - Birth weight
  - **MEI1-specific**:
    - Did you often suffer from physical problems as a child?
    - Did your umbilical cord fall off late after birth?
- **Congenital abnormalities**
- **Neonatal period**

**Developmental History**

- **Behavioural development**
  - Were milestones achieved on time?
- **Psychomotor development**
  - Were milestones achieved on time?
- **Speech & language development**
  - Were milestones achieved on time?

**Family History**

- **MEI1-specific**: Are there immune system problems in the family?
- **Pedigree of the family**
- **Siblings**
- **Father and his family**
- **Mother and her family**
- **Are parents consanguineous?**

**Physical Examination**

- **General appearance**
- **Length (SD)**
- **Weight (SD)**
- **Body Mass Index (BMI)**
- **Head circumference (SD)**
  - **MEI1-specific**:
    - Is there microcephaly?
    - Are there dysmorphic features?
    - Is there dwarfism or disproportionate growth?
- **Span length (span/length ratio)**
- **Facial appearance**
- **Thoracic**
- **Abdomen**
- **Spine**
- **Extremities**
- **Skin**

**Additional Immunological Testing (MEI1-specific)**

**Note**: Blood sampling in the morning (due to required tests)

- **Immunoglobulin levels**: IgG, IgG subclasses, IgA, IgM, IgD, IgE
- **Quick screen for autoantibodies**: ANA-screen
- **Extended immune phenotyping using four panels**:
  1. Screening immune phenotyping – overview of monocytes, neutrophils, etc.
  2. Extended TBNK – various lymphocyte subsets
  3. Memory B – detailed B-cell populations, including plasmablasts
  4. Plasma cell panel – focus on plasma cells using additional antibody-producing cell panel

**Required tubes**:

- **Serum tube**:
  - 2x S5 serum tube without gel (red, plastic, 5 mL)
- **EDTA tube**:
  - 2x K10 K2-EDTA tube (purple, 10 mL)

**Extended description of immune phenotyping subject_1**

| **Immunoglobulin levels** | | |
| --- | --- | --- |
| **Ig (sub)class** | **Conc. (g/L or U/ml)** | **Reference values** |
| **IgG** | 16.8 | 7-16 |
| **IgG1** | 11.1 | 4.9-11.4 |
| **IgG2** | 4.4 | 1.5-6.4 |
| **IgG3** | 0.76 | 0.2-1.1 |
| **IgG4** | 0.38 | 0.08-1.4 |
| **IgA** | 2.5 | 0.7-4.0 |
| **IgM** | 1.3 | 0.4-2.3 |
| **IgD** | <3 U/ml | <100 |
| **IgE** | 20 U/ml | <100 |
| **Immunoglobulin diagnostics** | | |
| **Parameter** | **Conc. (g/L)** | **Reference values / outcome** |
| **M-protein** | Absent | Absent |
| **ANA-screen**  **(auto-immune Ig)** | Negative | Negative |
| **Cell numbers** | | |
| **Cell type** | **Number (*10^9^/L)** | **Reference values** |
| **T-cell** | 1.23 | 0.70-2.10 |
| CD4+ T-Helper | 0.76 (62%) | 0.30-1.40 |
| CD8+ Cytotoxic | 0.38 (31%) | 0.20-0.90 |
| Regulatory | 0.05 (4%) | 0.02-0.09 |
| **B-cell** | 0.11 | 0.10-0.50 |
| Transitional | 0.005 (5%) | 0.003-0.050 |
| Naive | 0.055 (47%) | 0.057-0.447 |
| Central memory | 0.023 (20%) | 0.021-0.210 |
| IgM only | 0.004 (3%) | 0.013-0.122 |
| Class-switch | 0.019 (16%) |  |
| Plasmablasts | 0.003 (2%) | 0.001-0.023 |
| CD21low | 0.005 (5%) |  |
| **NK-cell** | 0.24 | 0.09-0.60 |
| Regulatory | 0.01 (4%) |  |
| Cytotoxic | 0.23 (96%) |  |

Table 1. Outcome of antibody analyses and immune cell evaluation

**FACS data**

**General sort**


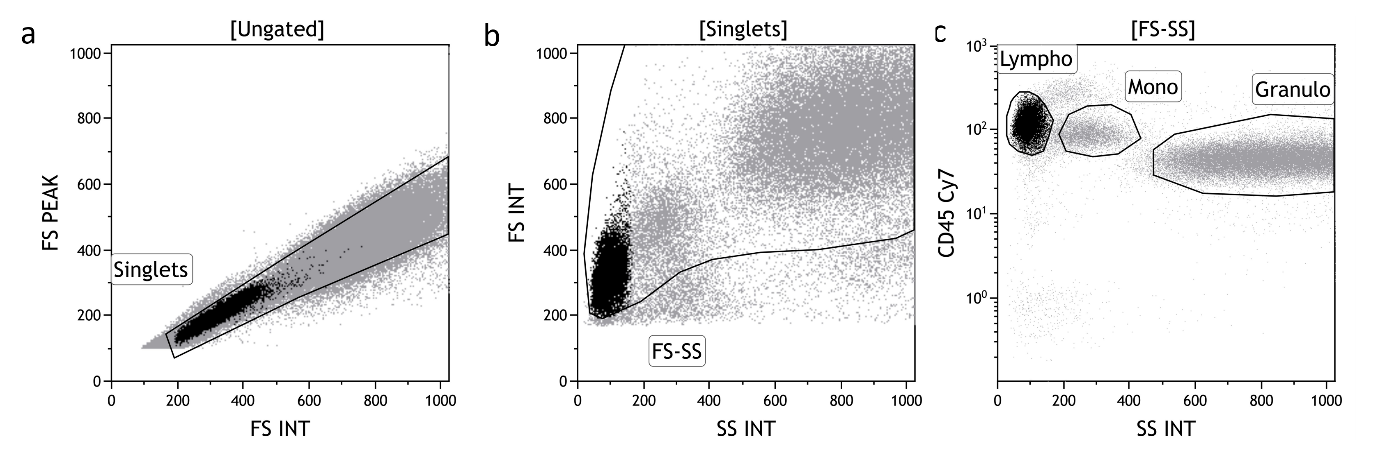


a) Based on FSC-INT and FSC-PEAK, doublets are excluded.

b) Based on FS and SS, live leukocytes are gated.

c) Lymphocytes are gated based on CD45^high^ and SS^low^ expression.

**B-cells**


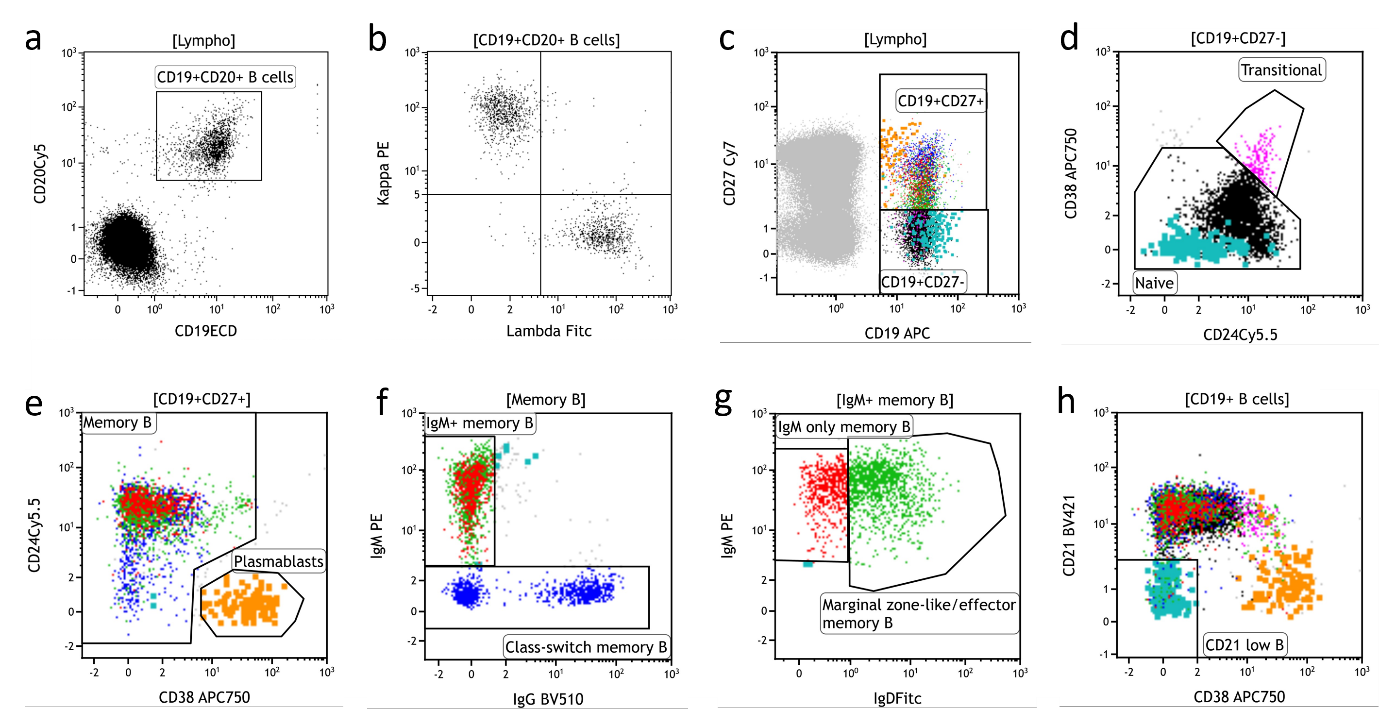


a) B-cells are gated based on CD19 and CD20 co-expression.

b) Within mature B-cells, the ratio of IgKappa/IgLambda expression is evaluated.

c) Within B-cells, CD27 expression is used to distinguish between activated and non-activated B-cells.

e) Within the CD27– B-cells, CD38 vs. CD24 expression distinguishes between naïve B-cells (black) and transitional B-cells (pink).

f) Within the CD27+ activated B-cells, CD24 vs. CD38 expression distinguishes between plasmablasts (orange) and memory B-cells.

g) Using IgM/IgG expression, memory B-cells can be further divided into IgM^+^ memory B-cells (green + red population) and class-switched B-cells (blue population).

h) Subsequently, based on IgM vs. IgD expression, one can distinguish between IgM-only memory B-cells (red population) and marginal zone-like/effector memory B-cells (green population).

i) The CD21^low^ cells (in turquoise) are defined based on CD21 and CD38 expression (CD19^+^CD21^-^CD38^-^).

**T-cells**

**
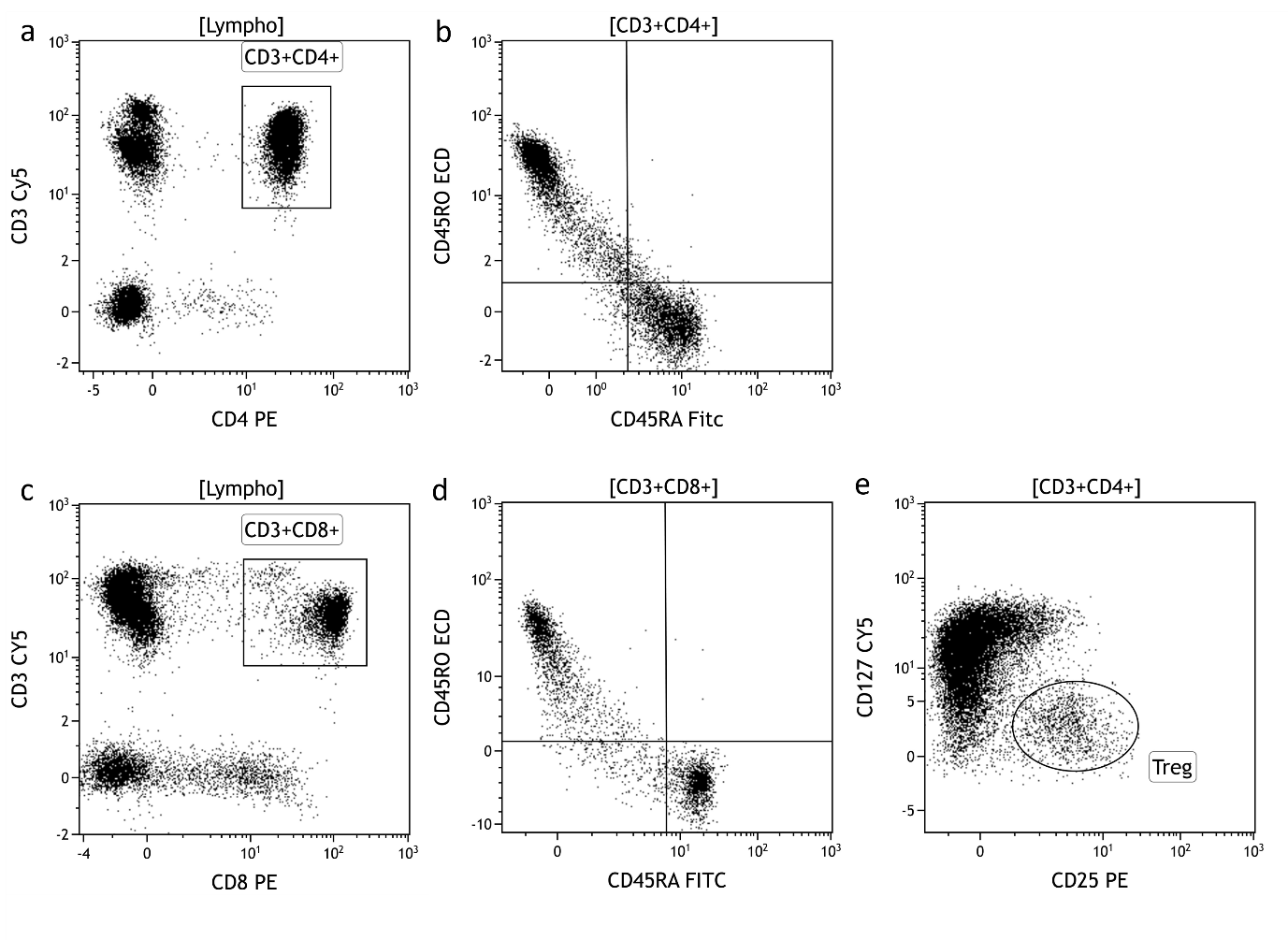
**

a) T-helper cells are gated based on CD3 and CD4 co-expression.

b) Within the T-helper cells, CD45RA/RO (ratio of naïve vs. effector/memory T-cells) is evaluated.

c) Cytotoxic T-cells are gated based on CD3 and CD8 co-expression.

d) Within the cytotoxic T-cells, CD45RA/RO (ratio of naïve vs. effector/memory T-cells) is evaluated.

e) Regulatory T-cells (Tregs) are gated based on CD127– and CD25+ expression within the CD3+CD4+ T-helper cell population.

**NK-cells**


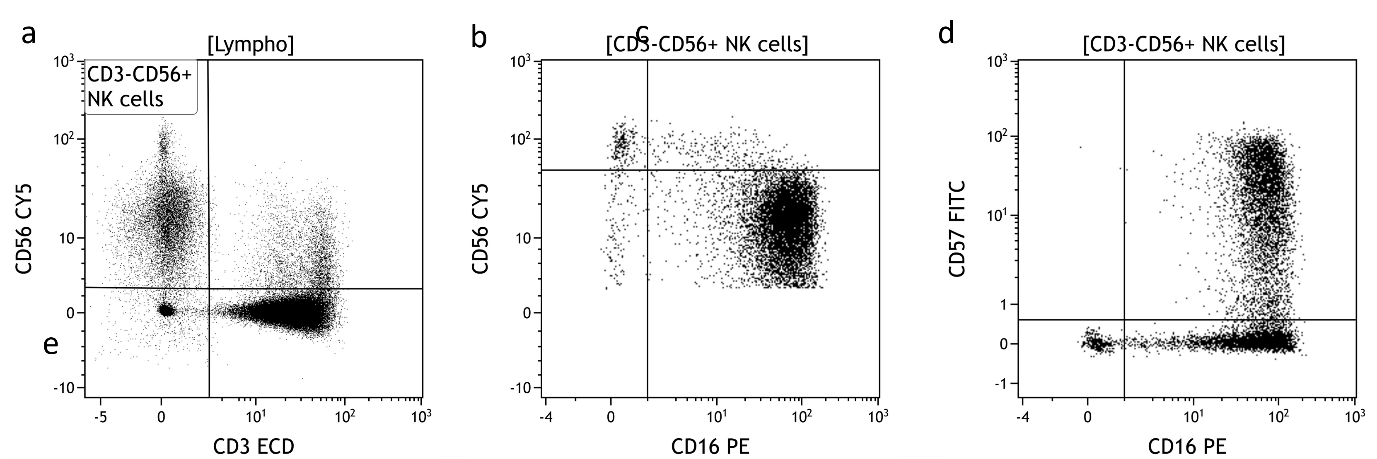


a) NK-cells are gated based on CD3– and CD56+ expression.

b) Within NK-cells, CD56++CD16– (regulatory) NK-cells and CD56+CD16+ (cytotoxic) NK-cells can be identified.

c) Within the cytotoxic NK-cells, CD57– and CD57+ NK-cell subpopulations can be distinguished.
