# Supplementary material for: A clinical protocol for the detection of comorbidities associated with monogenic causes of male infertility
Source: Hum Reprod. 2026 Mar 21;41(5):689–98. doi: 10.1093/humrep/deag038 (PMC13139667; doi:10.1093/humrep/deag038)
Supplement: deag038_Supplementary_Data_File_S8 [file deag038_supplementary_data_file_s8.docx]

Supplementary Data File S8

**DNAH17 phenotyping protocol and extended description of DNAH17 ophthalmic phenotyping**

*Completed by:*

*Date:*

**Phenotyping Male Infertility**

- **Date of consultation**
- **Study ID**
- **Age**

**Medical History**

- **Past medical history**
- **Current medication use**
- **Review of organ systems**

**Head/Hair**

- Skin, hair, nails: any abnormalities?

**Ears**

- Hearing

**Eyes**

- Vision
- **DNAH17-specific questions**:
  - Do you have problems with your vision?
  - Do you have any eye complaints?
  - Do you wear glasses or contact lenses?
  - Do you have difficulty seeing contrasts?
  - Are you color blind?
  - Do you have trouble distinguishing colors?
  - Do you suffer from night blindness?
  - Do your eyes move back and forth involuntarily?
  - Do you have trouble perceiving depth?
  - When you focus your vision on something, does the image tremble?

**Nose**

- Anosmia (loss of smell)

**Mouth**

- Tooth development (missing elements/cavities)

**Cardiological**

- Congenital heart defect, cardiomyopathy, palpitations, imaging

**Pulmonary**

- Respiratory infections, lung problems

**Digestive Tract**

- Gastrointestinal issues

**Liver**

- Jaundice, liver problems

**Urogenital tract**

- Congenital anomalies of the kidney and urinary tract
- Kidney problems and blood pressure
- Congenital anomalies of the internal and external reproductive organs

**Extremities**

- Abnormalities/extra fingers or toes

**Neurological Tract**

- Epilepsy
- Movement problems (balance/coordination)
- Muscle tone

**Endocrine Functions**

- Hormones other than sex hormones examined
- **DNAH17-specific**:
  - Do you have trouble remembering “lists”?

**Haematological System**

- Bruising, prolonged bleeding

**Skeletal Function**

- Hypermobility, growth abnormalities

**Skin**

- Eczema, hyper-/hypopigmentation

**Immune System**

**Social History**

- **Educational development**
- **Occupation**
- **Social status** (Note: do not assess presence of offspring)
  - Married, etc.: Registered partner
- **Involvement of social services or aids**
  - Assisted living? Extra support?

**Childhood History**

- **Birth history**
  - Pregnancy
  - Delivery
  - Gestational age
  - Birth weight
- **Congenital abnormalities**
- **Neonatal period**

**Developmental History**

- **Behavior**
- **Psychomotor development**
- **Speech & language development**
  - Were milestones achieved on time?
  - **DNAH17-specific**:
    - Were motor milestones achieved on time?
    - Did you find learning school material difficult?

**Family History**

- **Pedigree of the family**
- **Siblings**
- **Father and his family**
- **Mother and her family**
- **Are parents consanguineous?**

**Physical Examination**

- **General appearance**
- **DNAH17-specific**:
  - Signs of nystagmus?
- **Length (SD)**
- **Weight (SD)**
- **Body Mass Index (BMI)**
- **Head circumference (SD)**
- **Arm span (span/length ratio)**
- **Facial appearance**
- **Thoracic**
  - **DNAH17-specific**: Dextrocardia? Situs inversus?
- **Abdomen**
- **Spine**
- **Extremities**
- **Skin**

**Tests**

- **Eye tests**
  1. Refraction
  2. Visual acuity
  3. Axial length measurement
  4. Color vision test (HRR)
  5. Static perimetry (OD and OS)
  6. Dark adaptation test

Eye drops

- 1. Ophthalmological examination (fundoscopy)
  2. Optical Coherence Tomography (OCT) (including near-infrared reflectance) – mapping retinal layers
  3. Fundus photography
  4. If abnormalities in static perimetry: Microperimetry (OD and OS, mesopic, standard grid)
  5. Electroretinogram (ERG) – mapping electrical activity of photoreceptors and bipolar cells
     - Scotopic (dark-adapted)
     - Photopic
     - mfERG

12. Adaptive Optics (AO) – mapping cone photoreceptor organization in the retina

If indicated:

13. MRI

14. Torso ultrasound

**Extended description of ophthalmic test results subject_2**

**Refraction:**
Note that the patient has a history of refractive laser surgery, which may influence current refractive status. Nonetheless, the measured values are stable and within expected postoperative limits.

**Visual Acuity Testing:**
No evidence of visual impairment was observed; on the contrary, the patient's performance was better than expected for their age group.

**Axial Length Measurement:**
Axial length measurements were within expected normative values.

**Color Vision Testing (HRR):**
Color discrimination was normal. The patient was able to correctly identify all plates, indicating no red-green or blue-yellow color vision deficiency.

**Static Perimetry (OD and OS, OP3 / 32 Dynamic White-White):**
Visual field testing revealed sensitivity within the normal ranges across the tested field for both eyes. No scotomas or peripheral defects were detected.

**Fundoscopy:**
Direct examination of the retina showed a healthy optic nerve head, clear macula, and normal retinal vessels. No signs of retinal pathology, hemorrhages, or degenerative changes were noted.

**Fundography left eye Fundography right eye**


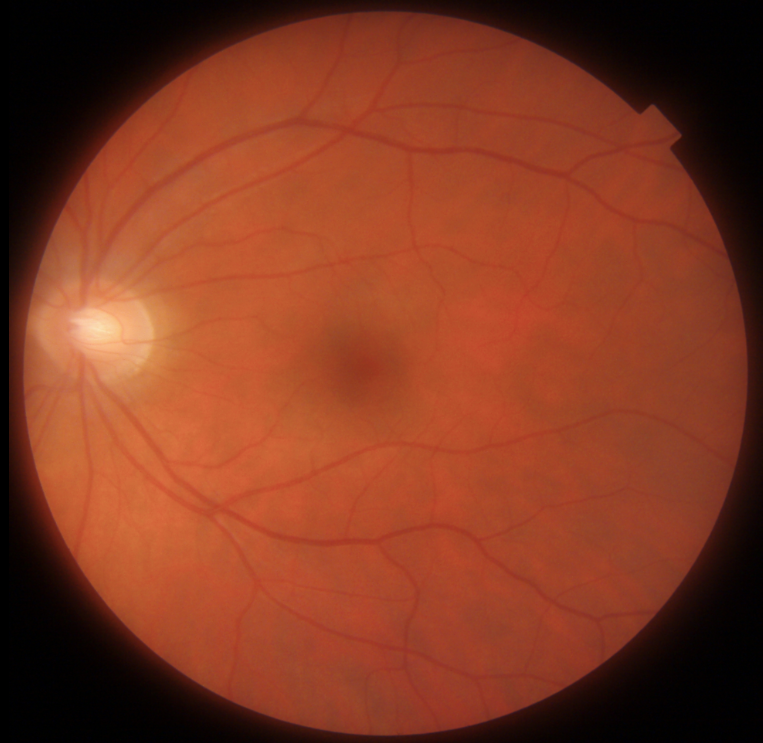

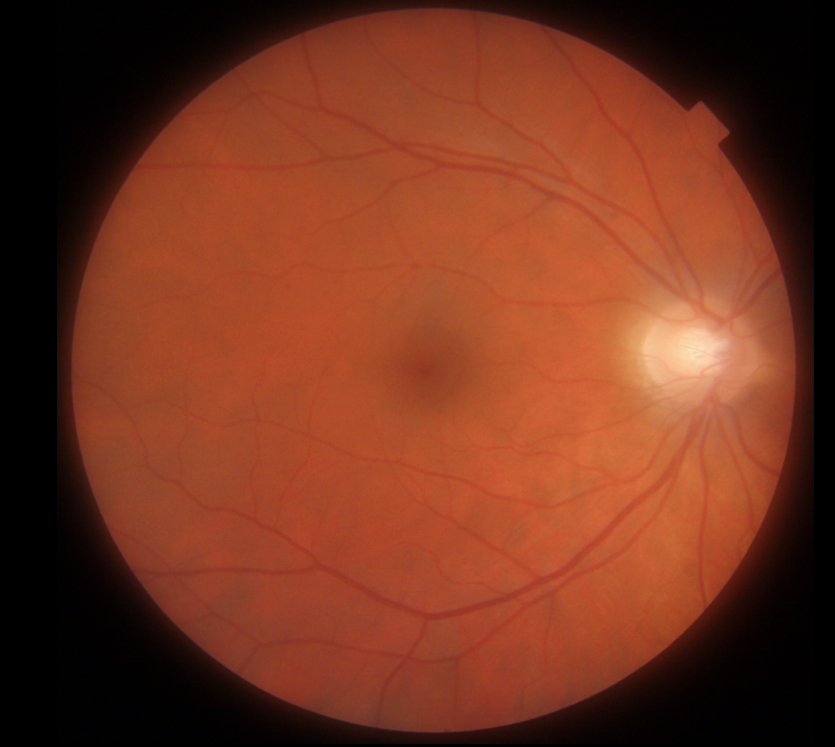


**Optical Coherence Tomography (OCT) (including near-infrared reflectance):**
OCT showed normal architecture of the retinal layers. Retinal thickness values were within normal limits, and no signs of edema, atrophy, or abnormal reflectivity were present.

**Fundus Photography (Topcon):**
High-resolution images confirmed a healthy appearance of the retina, optic disc, and macular region. No drusen, pigmentary changes, or vascular abnormalities were observed.

**Dark Adaptation Test:**
The response to darkness showed a normal adaptation curve, indicating healthy rod photoreceptor function. There were no delays or abnormalities in the recovery of visual sensitivity in low light.

**Electroretinogram (ERG):**

**Scotopic (V5):** Rod-mediated responses under dark-adapted conditions were normal in amplitude and timing.

**Photopic (V6.1):** Cone-mediated responses were within physiological norms, with no indication of cone dysfunction.

**mfERG (61 Hex):** Multifocal responses from the central retina were uniform and symmetric, with amplitudes and implicit times within the normal range.

**mfERG**


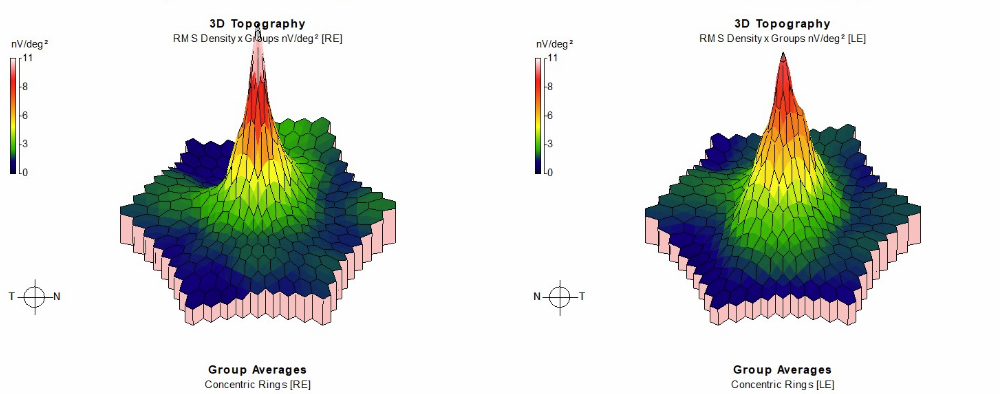


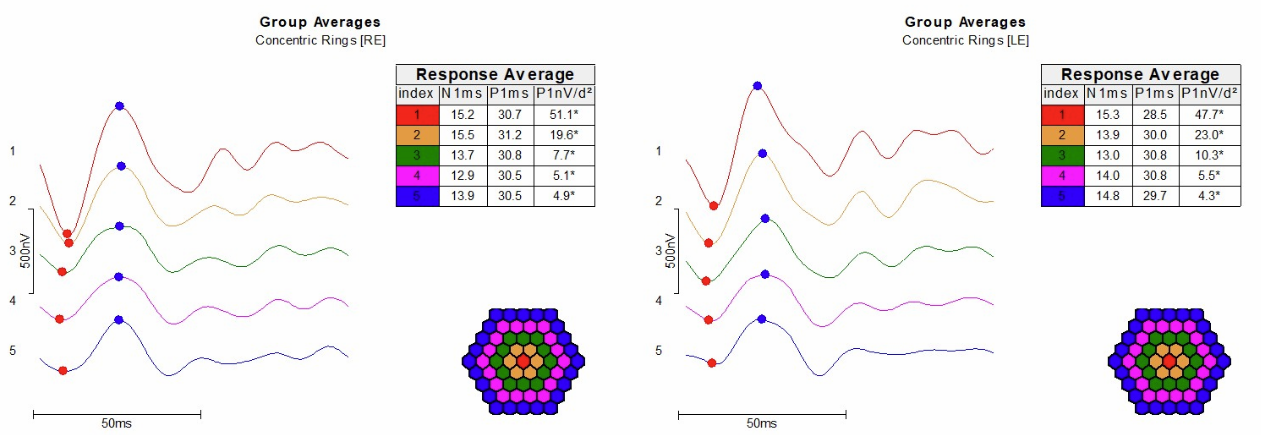


**Adaptive Optics (AO):**
Imaging of the cone photoreceptor mosaic showed regular spacing and density consistent with a healthy retina. No signs of cone loss or disorganization were present.
